# Combined transcriptomic and ChIPseq analyses of the *Bordetella pertussis* RisA regulon

**DOI:** 10.1128/msystems.00951-23

**Published:** 2024-03-12

**Authors:** Loïc Coutte, Rudy Antoine, Stephanie Slupek, Camille Locht

**Affiliations:** 1 U1019–UMR9017, University of Lille, CNRS, Inserm, CHU Lille, CIIL-Center for Infection and Immunity of Lille, Institut Pasteur de Lille, Lille, France; University of Wisconsin-Madison, Madison, Wisconsin, USA

**Keywords:** *Bordetella pertussis*, RNAseq, ChIPseq, RisA

## Abstract

The regulation of *Bordetella pertussis* virulence is mediated by the two-component system BvgA/S, which activates the transcription of virulence-activated genes (*vag*s). In the avirulent phase, the *vag*s are not expressed, but instead, virulence-repressed genes (*vrg*s) are expressed, under the control of another two-component system, RisA/K. Here, we combined transcriptomic and chromatin immunoprecipitation sequencing (ChIPseq) data to examine the RisA/K regulon. We performed RNAseq analyses of RisA-deficient and RisA-phosphoablative *B. pertussis* mutants cultivated in virulent and avirulent conditions. We confirmed that the expression of most *vrg*s is regulated by phosphorylated RisA. However, the expression of some, including those involved in flagellum biosynthesis and chemotaxis, requires RisA independently of phosphorylation. Many RisA-regulated genes encode proteins with regulatory functions, suggesting multiple RisA regulation cascades. By ChIPseq analyses, we identified 430 RisA-binding sites, 208 within promoter regions, 201 within open reading frames, and 21 in non-coding regions. RisA binding was demonstrated in the promoter regions of most *vrg*s and, surprisingly, of some *vag*s, as well as for other genes not identified as *vag*s or *vrg*s. Unexpectedly, many genes, including some *vag*s, like *prn*, *brpL*, *bipA,* and *cyaA*, contain a BvgA-binding site and a RisA-binding site, which increases the complexity of the RisAK/BvgAS network in *B. pertussis* virulence regulation.

**IMPORTANCE:**

The expression of virulence-activated genes (*vag*s) of *Bordetella pertussis*, the etiological agent of whooping cough, is under the transcriptional control of the two-component system BvgA/S, which allows the bacterium to switch between virulent and avirulent phases. In addition, the more recently identified two-component system RisA/K is required for the expression of *B. pertussis* genes, collectively named *vrg*s, that are repressed during the virulent phase but activated during the avirulent phase. We have characterized the RisA/K regulon by combined transcriptomic and chromatin immunoprecipitation sequencing analyses. We identified more than 400 RisA-binding sites. Many of them are localized in promoter regions, especially *vrg*s, but some were found within open reading frames and in non-coding regions. Surprisingly, RisA-binding sites were also found in promoter regions of some *vag*s, illustrating the previously underappreciated complexity of virulence regulation in *B. pertussis*.

## INTRODUCTION

Productive host-bacterial pathogen interactions almost invariably require fine-tuned gene regulation mechanisms to ensure that the bacterial virulence factors are produced at the appropriate time and place. This generally occurs through sophisticated transcriptional regulatory circuits, the detailed understanding of which may be helpful for the design of novel therapeutic and/or prophylactic approaches against bacterial infections. The production of virulence factors by *Bordetella pertussis*, the etiological agent of whooping cough, is tightly controlled at the transcriptional level by the two-component system BvgA/S (1). BvgS is a sensor kinase that, in its active form, dimerizes, autophosphorylates, and then transfers the phosphoryl group to the response regulator BvgA. Phosphorylated BvgA activates the transcription of the *B. pertussis* virulence genes, collectively called *vag*s, which include genes coding for adhesins, such as filamentous hemagglutinin (*fha*) and fimbriae (*fim2* and *fim3*), and toxins, including pertussis toxin (*ptx*), adenylate cyclase toxin (*cya*), and dermonecrotic toxin (*dnt*), as well as other virulence factors. The *vag*s represent approximately 3% of the *B. pertussis* gene content, but their transcripts represent more than 10% of the total transcripts (2).

The signals that activate BvgA/S within the infected host are not known, but it is possible that, unlike most bacterial two-component systems, BvgA/S is constitutively activated in *B. pertussis* in the virulence phase (3). In the laboratory, the BvgA/S system can be switched off by growing the organism in the presence of chemical modulators, such as MgSO_4_ (4). While under these modulating conditions, the expression of the *vag*s is turned off, and the expression of a different set of genes, collectively called *vrg*s, is activated. The expression of most *vrg*s is under the control of another two-component system, named RisA/K (5, 6). Most *vrg*s require phosphorylated RisA, catalyzed by RisK, and the presence of c-di-GMP for their transcription. However, some are activated by RisA in its non-phosphorylated form in the presence or absence of c-di-GMP (5), implying that the mechanism of RisA-dependent regulation is much more complex than that of BvgA-dependent regulation. c-di-GMP can be hydrolyzed by the phosphodiesterase encoded by the *vag bvgR* (7), which thereby reduces *vrg* expression in non-modulating conditions.

The *vrg*s also represent approximately 3% of the total *B. pertussis* gene content, but, unlike those of the close relative *Bordetella bronchiseptica*, many of the *B. pertussis vrg*s are pseudogenes (2), and their function in pertussis pathogenesis remains unknown. However, it has been hypothesized that the *vrg*s may be important for *B. pertussis* transmission (8).

While the BvgA/S regulon has been extensively studied using transcriptomic approaches and chromatin immunoprecipitation sequencing (ChIPseq), much less is known about the RisA/K regulon. Using a microarray approach, we have previously shown that RisA-regulated genes can be sub-divided into four different categories: those that require phosphorylated RisA and the presence of c-di-GMP, those that require phosphorylated RisA, but do not require c-di-GMP, those that require c-di-GMP, but do not require RisA to be phosphorylated, and those that require RisA in the absence of phosphorylation and c-di-GMP (5). Here, we used RNAseq analysis to confirm and extend these findings by identifying several RisA-regulated small non-coding RNAs, which could not be identified by microarray studies. In addition, we performed ChIPseq analyses to identify directly and indirectly RisA-activated *vrg*s, as defined by their upregulation under modulating conditions. Surprisingly, the ChIPseq analyses revealed *vag*s containing both RisA- and BvgA-binding sites in their promoter, suggesting a complex regulation mechanism of these genes.

## RESULTS

### RNAseq analysis of the RisA regulon

The *B. pertussis* Tohama I derivative BPSM (9), BPSM*∆risA* (5), and BPSM*risA*^D60N^, a BPSM derivative producing a phosphoablative RisA analog, were grown in modulating, i.e., in the presence of 50 mM MgSO_4_ (m), or non-modulating conditions (nm), and their total transcriptome was analyzed by RNAseq. We arbitrarily considered a Log_2_ fold change (Log_2_FC) of <−3 or >3 to identify RisA-regulated and/or MgSO_4_-modulated genes. In contrast to our microarray studies performed previously (5), RNAseq analyses allowed us to obtain the absolute quantification of gene expression levels and provided a wider amplitude of gene regulation than microarray studies, ranging from Log_2_FC −10.52 to 6.65 and −8.67 to 6.40, respectively.

Here, we used modulation to distinguish between *vag*s and *vrg*s, respectively, repressed and activated in the presence of MgSO_4_. Since the presence of MgSO_4_ switches off BvgA/S-regulated genes, including *bvgR*, likely coding for a c-di-GMP phosphodiesterase (7), c-di-GMP levels are elevated by modulation with MgSO_4_. As a co-factor of RisA, c-di-GMP is required for the activation of most RisA-regulated genes. Therefore, the effect of RisA phosphorylation on *vrg* expression may best be seen in modulating conditions, although some genes may require phosphorylated RisA independently of c-di-GMP. Conversely, the modulation of *vag*s may or may not depend on RisA.

The previous microarray study identified six clusters of genes: *vag*s that are modulated by MgSO_4_ (cluster 1), *vag*s for which the modulation depends on RisA (cluster 2), RisA-induced but non-modulated genes (cluster 3), RisA-repressed genes not regulated by modulation (cluster 4), genes repressed by modulation and not regulated by RisA (cluster 5), and *vrg*s that are regulated by RisA and modulation (cluster 6). Overall, the RNAseq data were consistent with those of our previous microarray study (5) and confirmed these six clusters. However, the higher sensitivity of the RNAseq allowed us to better define these clusters using a higher stringency with Log_2_FC of <−3 or > 3 (Table S1). For cluster one, the RNAseq analyses identified 75 *vag*s, including the 58 *vag*s previously identified in the microarray study. For cluster 2, the RNAseq data identified 21 genes, including the 11 previously identified genes. For clusters 3, 4, 5, and 6, the RNAseq data identified 27, 84, 6, and 69 genes, respectively, compared to 3, 9, 4, and 33 genes, respectively, in the microarray analysis.

**Fig 1:**
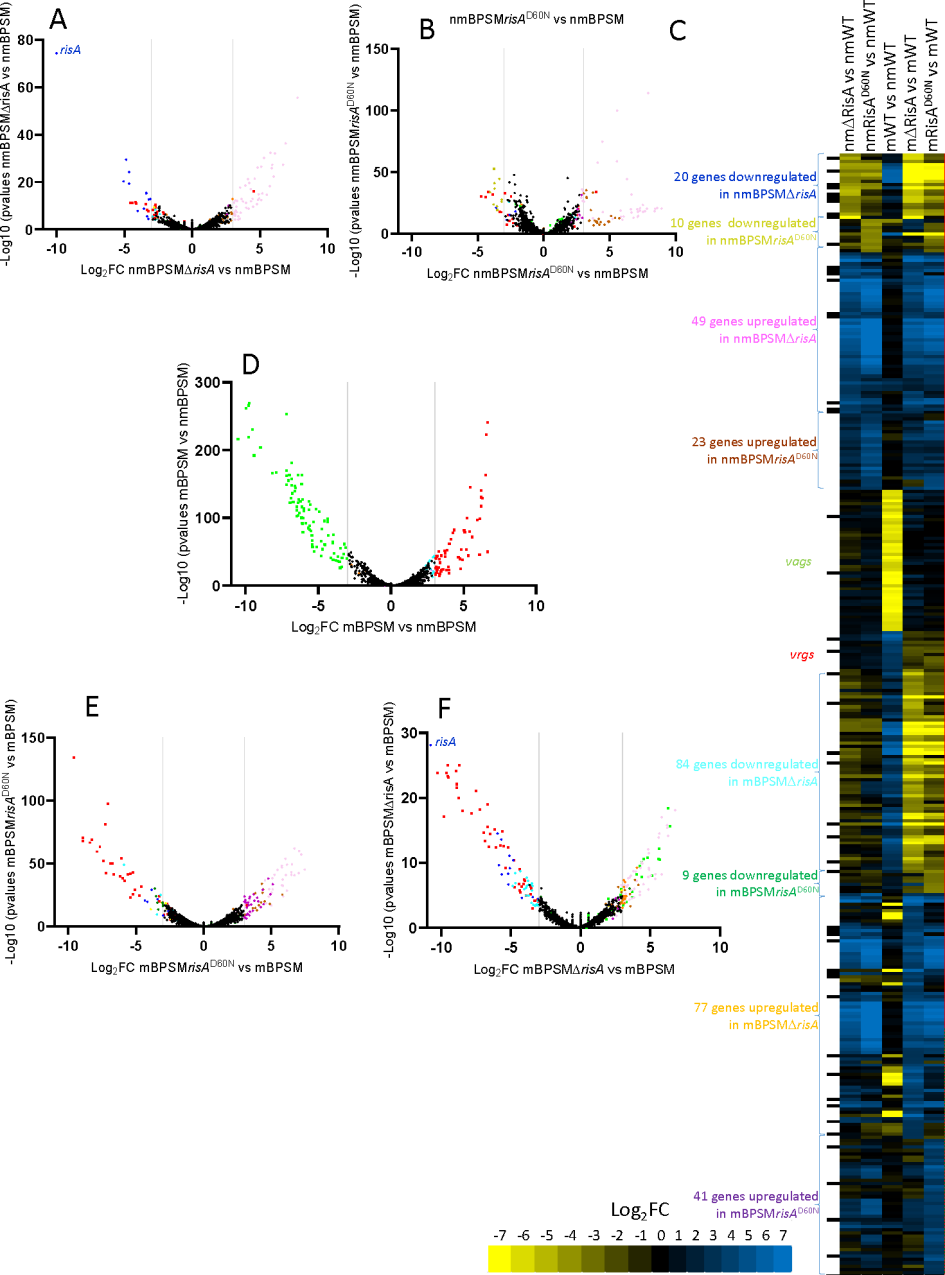
Regulation of genes by RisA and modulation. Volcano plots showing the regulation of the genes in nmBPSMΔ*risA* vs nmBPSM (**A**), nmBPSMrisA^D60N^ vs nmBPSM (**B**), mBPSM vs nmBPSM (**D**), mBPSMrisAD60N vs mBPSM (**E**), and mBPSMΔrisA vs mBPSM (**F**). The vertical gray bars represent the cutoff used (Log_2_FC −3 or +3). Genes were clusterized and are presented in different colors according to their regulation presented on the heat map (**C**). On the heat map, the first column (black boxes) shows the presence of RisA-binding sites in the promoter regions. The following columns present heat maps of the gene regulation comparing nmBPSMΔrisA vs nmBPSM (nmDRisA vs nmWT), nmBPSMrisA^D60N^ vs nmBPSM (nmRisA^D60N^ vs nmWT), mBPSM vs nmBPSM (mWT vs nmWT), mBPSMΔrisA vs mBPSM (mDRisA vs mWT), and mBPSMrisA^D60N^ vs mBPSM (mRisA^D60N^ vs mWT).

In nm conditions, 20 genes required the presence of RisA, as they were less expressed in nmBPSM∆*risA* than in nmBPSM, 10 were less expressed in nmBPSMrisA^D60N^ (Fig. 1A through C; Tables S2 and S3), confirming our previous findings (5) that for some genes RisA regulation occurs independently of its phosphorylation. The expression of 23 genes was upregulated by phosphoablative RisA^D60N^ (Fig. 1B; Tables S2 and S3), while 49 genes were upregulated in the absence of RisA.

**Fig 2:**
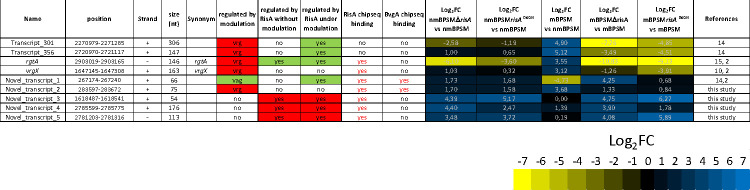
Regulated putative sRNA identified by *B. pertussis* BPSM RNAseq analysis. Transcriptional start and stop sites are the first and last nucleotides of the detected putative sRNA relative to the *B. pertussis* Tohama I BX470248 genome annotation. Strands “+” or “−” represent forward and reverse directions, respectively, to the orientation of the *B. pertussis* Tohama I BX470248 genome annotation. Synonyms correspond to previously described sRNA. RisA binding corresponds to this study, while BvgA binding is from Coutte et al. (2). Log_2_FC corresponds to the Log_2_ fold change of the RPKM of the detected putative sRNA in the tested conditions. nt, nucleotides.

In modulating conditions, the expression of 84 genes required the presence of RisA (Fig. 1D and E), and the activation of 9 genes required phosphorylated RisA, as they were specifically less expressed in mBPSMrisA^D60N^ than in mBPSM (Fig. 1E and F). Among the 77 genes for which expression was increased by the absence of RisA, some, like the *vag*s coding for adhesine production and maturation (*sphB1, prn, fhaB-fhaC*, and *fim2*), the toxin adenylate cyclase (*cyaA*), a methyltransferase (*bp2936*), and a ferrisiderophore receptor (*bfrD*), showed stronger modulation in the BPSM than in the BPSM*∆risA* background, indicating that their modulation depends on the presence of RisA (Fig. 1D and E; Table S1, cluster 2; Table S4), as shown previously (5). The expression of 24 *vag*s was no longer modulated in mBPSM*∆risA*, while it was modulated in mBPSM (Table S4). The expression of 41 genes was upregulated by RisA in its non-phosphorylated form, as their expression was stronger in mBPSM*risA*^D60N^ than in mBPSM (Fig. 2E and F). They comprise genes that show the same feature in nm conditions.

Overall, RisA is involved in the regulation of 150 genes according to its state of phosphorylation and modulation, including *vag*s, almost all the *vrg*s, and genes that are not regulated by modulation. Additionally, BPSM*risA*^D60N^ presented a Log_2_FC over BPSM very close to the pre-defined cutoff, independent of modulation (Fig. 1B and F; yellow, brown, dark green, and purple dots), suggesting that some genes regulated by RisA^D60N^ were only “partially regulated,” as differential regulation was much stronger when BPSM was compared to BPSM∆*risA* than when it was compared to BPSM*risA*^D60N^, both in modulating and non-modulating conditions.

### Putative sRNA regulated by RisA

To search for RisA-regulated non-coding transcripts, we ran the RNAseq data through Rockhopper, which identified 446 regions not related to previously annotated open reading frames (ORFs). Among them, 189 were regulated by RisA and/or modulation in at least one of the tested conditions with a Log_2_FC > 3 or <−3 (Table S5). Most of them are 3′ or 5′ untranslated regions (UTR) or intergenic regions of operons. However, nine transcripts are not 3′ or 5′ UTR and may be putative RisA-regulated sRNA (Fig. 2). The ncRNAs starting with "transcript" were already predicted in a previous study by Amman F *et al.* (RNA Biol 2018), while the ncRNAs starting with "novel_transcript" were identified with our datasets of RNAseq in this study and a previous study by Coutte L *et al.* (mSystems, 2020). The expression of Transcript_301, Transcript_356, *rgtA,* and *vrgX* was enhanced in mBPSM compared to nmBPSM but strongly reduced in mBPSM*∆risA* and mBPSM*risA*^D60N^ and therefore presents a *vrg*-like profile of expression (Fig. S1 to S4). Novel_transcript_1 was downregulated in mBPSM compared to nmBPSM and upregulated in mBPSM*∆risA* but not in mBPSM*risA*^D60N^, indicating a *vag* profile of regulation. It is located near the 5′ end of *bp0258* (Fig. S5), a gene that was not regulated in any of the tested conditions. Novel_transcript_2 is located in the diverging promoter region between *bp0277* and *bp0278* (Fig. S6). While the expression of *bp0277* and *bp0278* was not regulated in any of the tested conditions, the expression of Novel_transcript_2 was increased in mBPSM, mBPSMΔ*risA,* and mBPSM*risA*^D60N^ compared to nmBPSM (Fig. 2; Fig. S6). Novel_transcript_3 was not regulated by modulation. However, its expression was increased in BPSMΔ*risA* and BPSM*risA*^D60N^ in modulating and non-modulating conditions (Fig. 2). It is located in a non-annotated region of the *B. pertussis* chromosome between *bp1545A* and *bp1546* (Fig. S7). Novel_transcript_4 is located in the 5′ UTR of the *vrg bp2629* (Fig. S8). However, it is transcribed in the opposite orientation to *bp2629,* and its expression was increased in BPSMΔ*risA* and BPSM*risA*^D60N^ in modulating and non-modulating conditions compared to BPSM, although the increase did not reach a Log_2_FC > 3 in BPSM*risA*^D60N^ (Fig. 2). Finally, Novel_transcript_5 is located in the 5′ UTR of *bp2626*, in the diverging promoter region between *bp2625* and *bp2626* and transcribed in the opposite orientation to *bp2626* (Fig. S9). *bp2625* is not regulated in any of the tested conditions. *bp2626* was very weakly transcribed with an RPKM (reads per kilobase of transcript per million reads mapped) of 15, while Novel_transcript_5 shows an RPKM of 475 in the same condition. This transcript was more expressed in modulated and non-modulated BPSM*ΔrisA* and BPSM*risA*^D60N^ than in BPSM (Fig. 2).

### RisA a regulator of regulators

**Fig 3:**
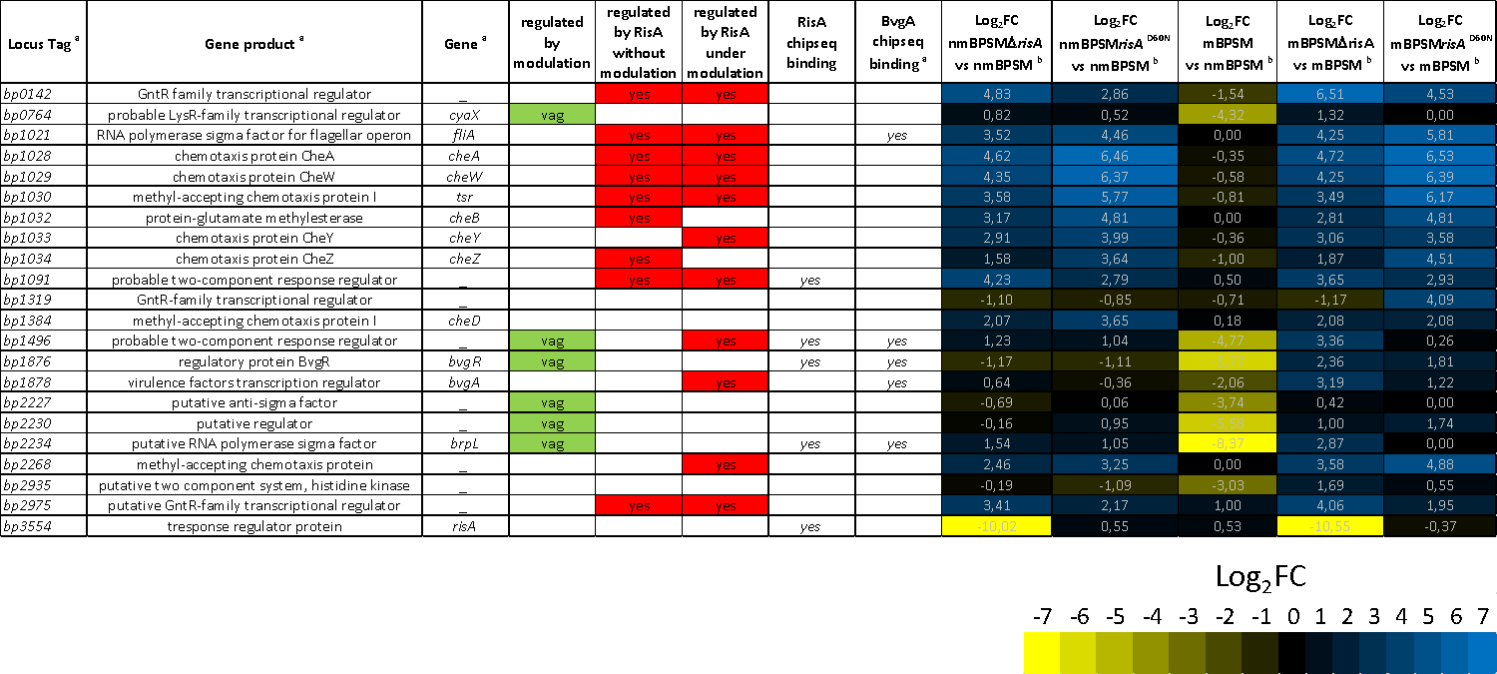
Regulation of genes coding for putative *B. pertussis* regulators. Genes and gene products correspond to the *B. pertussis* Tohama I BX470248 genome annotation. Log_2_FC corresponds to the Log_2_ fold change of the RPKM of the gene in the tested conditions. RisA binding corresponds to this study, while BvgA binding is from Coutte et al. (2).

Among the RisA-regulated genes, 22 genes encode proteins with putative regulatory functions, suggesting a RisA regulation cascade (Fig. 3). Six of them are *vag*s, coding for the adenylate cyclase regulator CyaX*,* the phosphodiesterase BvgR*,* a putative anti-sigma factor (BP2227)*,* the regulator of the type III secretion system BrpL, and two regulators of unknown function (BP1496 and BP2230). *bp1319,* coding for a regulator of unknown function, was more expressed in mBPSM*risA*^D60N^ than in the other conditions. Twelve genes were more expressed in BPSMΔ*risA* and BPSM*risA*^D60N^ than in BPSM in both modulating and non-modulating conditions, suggesting a RisA-mediated regulation of this gene. Among them, *bp0142* expression was stronger in BPSMΔ*risA* than in BPSM*risA*^D60N^ in both modulating and non-modulating conditions. As expected, *risA* was not expressed in nmBPSMΔ*risA* and mBPSMΔ*risA* (Fig. 1A and D), while the *risA*^D60N^ allele was expressed in nmBPSM*risA*^D60N^ and mBPSM*risA*^D60N^ at the same level as *risA* in BPSM in the same conditions.

### ChIPseq analysis of RisA binding in *B. pertussis*

**Fig 4:**
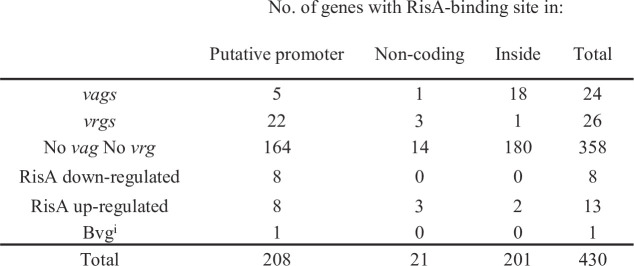
Summary of ChIPseq analysis of the *B. pertussis* RisA regulon. Putative promoter regions are located between two ORFs orientated in opposite directions. Non-coding regions are not annotated in the *B. pertussis* Tohama I BX470248 genome. Inside means within annotated ORF of the *B. pertussis* Tohama I BX470248 genome. *vag*s present a Log_2_FC < −3 in the mBPSM RNAseq analysis. *vrg*s present a Log_2_FC > 3 in the mBPSM RNAseq analysis. No *vag* No *vrg* are genes not identified as *vag* or *vrg* in the RNAseq analysis. RisA downregulated genes present a Log_2_FC < −3 in the nmBPSMΔ*risA* RNAseq analysis. RisA upregulated genes present a Log_2_FC of >3 in the nmBPSMΔ*risA* RNAseq analysis. Bvg^i^ corresponds to *bipA*, not regulated by modulation under the conditions tested here but requiring BvgA, as described by Coutte et al. (5).

ChIPseq analyses were performed to localize RisA-binding sites on the genome of *B. pertussis*. Among the signals identified by CLC genomics peak caller using all 10 data sets (two for each nmBPSM, mBPSM, nmBPSM*∆risA*, nmBPSM*risA*^D60N^, and mBPSM*risA*^D60N^), we obtained 2,792 peaks with a peak shape score > 5 and an associated *P* value comprised between 2.92 × 10^−7^ and 1.07 × 10^−232^ (Table S6), which revealed 430 RisA-binding sites distant from each other by at least 150 bp (Fig. 4).

None of the RisA-binding sites were detected in BPSMΔ*risA*, indicating the specificity of the assay. Most of them were detected in BPSM*risA*^D60N^, indicating that RisA may bind to its cognate DNA sites even in its non-phosphorylated state. However, for some ChIPseq clusters (73, 83, 90, 94, 134, 156, 184, 231, 233, 269, 279, 281, 301, 332, 358, 365, 396, and 429), binding sites were detected only in BPSM (Table S6) and not in BPSM*risA*^D60N^, indicating the importance of RisA phosphorylation for binding to these sites. For each RisA-binding site, the location coordinate was defined as the mean of the coordinates of the center of the RisA ChIPseq peak found in each condition. The resulting RisA-binding site locations were clustered in three different categories.

**Fig 5:**
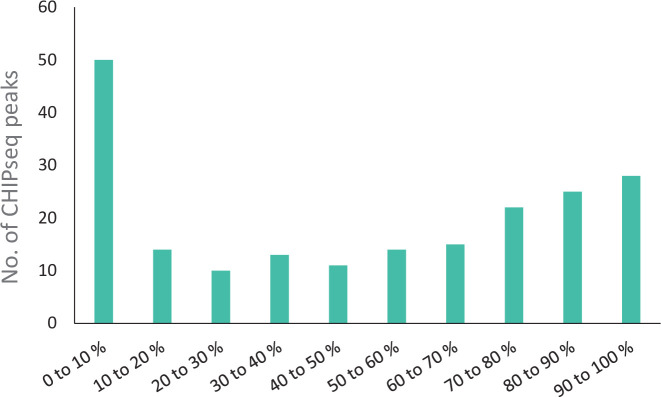
Localization of the RisA-binding sites within ORFs. ChIPseq peaks are depicted according to the localization within the ORF and are expressed as percentages of the total length of the corresponding ORF.

For the first category, 208 RisA-binding sites were found in putative promoter regions of annotated ORFs (Fig. 4; Table S7; as examples, see *vrg bp1736* and *vag prn* promoters in Fig. S10 and S11, respectively). Putative promoter regions were defined as regions upstream of an ORF spanning between −1 and −1,582 bp from the predicted translational start site and not overlapping with an adjacent ORF. Most RisA-binding sites of this category are located close to the predicted ATG translational start site (Fig. S12). Among the RisA-binding sites of this category, 58 were found in the putative promoter regions located between two ORFs orientated in opposite directions. The second category comprises 21 RisA-binding sites in non-coding regions not located in the proximity of annotated ORFs (Table S6). The second largest category is the third category with 201 RisA-binding sites found within annotated ORFs (Fig. 4; for example, see *bp0188* in Fig. S13). The distribution of the RisA-binding sites within ORFs shows a predominance of sites within the first 10% and last 10% of the ORFs with, respectively, 50/201 and 28/201 RisA-binding sites and could therefore be part of the promoter/operator region of the selected gene or downstream gene, respectively (Table S6; Fig. 5). Scatter plots showing the reproducibility between biological ChIPseq replicates and the coverage across strains and conditions are presented in Fig. S14 and S15.

### Integration of transciptomic and ChIPseq data

The RNAseq data from this study together with the data from the microarray analysis (5) identified 75 *vrg*s, corresponding to clusters 5 and 6 (Table S1), grouped into 35 transcriptional units. A combination with the ChIPseq data indicates that 19 of them have a RisA-binding site in their putative promoter region (Table S8), including two within an adjacent ORF but very close to the ATG starting site. Among the 16 remaining *vrg* transcriptional units, RisA-binding sites were found in promoter regions of *bp2090, bp2157, bp2731*, and *bp3710*, but with reads just under the pre-determined cutoff of 1,000 reads (974 reads, 890 reads, 792 reads, and 892 reads, respectively, Table S8). For *fim3*, identified as *vrg*, a RisA-binding site was not identified in its promoter region but in the promoter region of the upstream non-coding region called *vrgX* that was shown to regulate *fim3* expression (10). Thus, among the identified 35 *vrg*s transcriptional units regulated by RisA, 12 did not have a RisA-binding site in proximity, suggesting indirect regulation of these genes by RisA (Table S8). Moreover, in addition to *vrgX*, two further non-coding sRNA, *rgtA* and putative Novel_transcript_2, contain a RisA-binding site and present a *vrg*-like profile of regulation.

The 96 *vag*s and the Bvg^i^ gene *bipA*, corresponding to clusters 1 and 2 (Table S1), can be grouped into 43 transcriptional units (Table S9). While most of them do not contain a RisA-binding site in their promoter region, surprisingly, some genes coding for virulence factors involved in adhesion, type III secretion, and resistance to serum killing (*prn*, *bipA*, *bp1496*, *fimB*, *brpL,* and *vag8*) do contain a RisA-binding site in their putative promoter region (Fig. 4; Table S9). A RisA-binding site was also found within the ORFs of *bp0400* and *bp1875*, upstream of the adjacent *vag*s *bp0398-bp0399* and *bvgR*, respectively. A RisA-binding site was found in promoter regions of adenylate cyclase-encoding gene *cyaA* but with reads just under the pre-determined cutoff of 1,000 reads (885 reads). In addition, we detected a RisA-binding site in the upstream region of putative Novel_transcript_1, which also presents a *vag*-like profile of regulation. With the exception of *vag8*, the regulation by modulation of all these *vag*s and putative sRNA was modified in mBPSMΔ*risA* compared to mBPSM. Curiously, 14 RisA-binding sites were found within the ORF of *vag*s, corresponding to eight transcriptional units (Fig. 5; Table S9).

Among the 72 genes that were upregulated in nmBPSM*ΔrisA* and/or nmBPSM*risA*^D60N^ compared to nmBPSM, 10 contain a RisA-binding site. Among the 30 genes that were downregulated in nmBPSMΔ*risA* and/or nmBPSM*risA*^D60N^ compared to nmBPSM, 11 contain a RisA-binding site. With two exceptions, all these sites were located in putative promoter regions (Table S2)

Finally, 358 RisA-binding sites were found close to or within ORFs that were not regulated by modulation or RisA. Among them, 164 are located in putative promoter regions, 180 within ORFs, and 14 in non-annotated regions.

Among the 301 genes annotated in the *B. pertussis* genome that code for proteins with a putative regulatory function, 27 present a RisA-binding site (Table S10) and may therefore be candidates in the RisA regulon cascade. In the tested conditions, only three of them are regulated by modulation (*bp1496, bvgR,* and *brpL*), and the expression of one (*bp1091*) is repressed by RisA.

### Analysis of the *prn* promoter region by 5′ RACE

**Fig 6:**
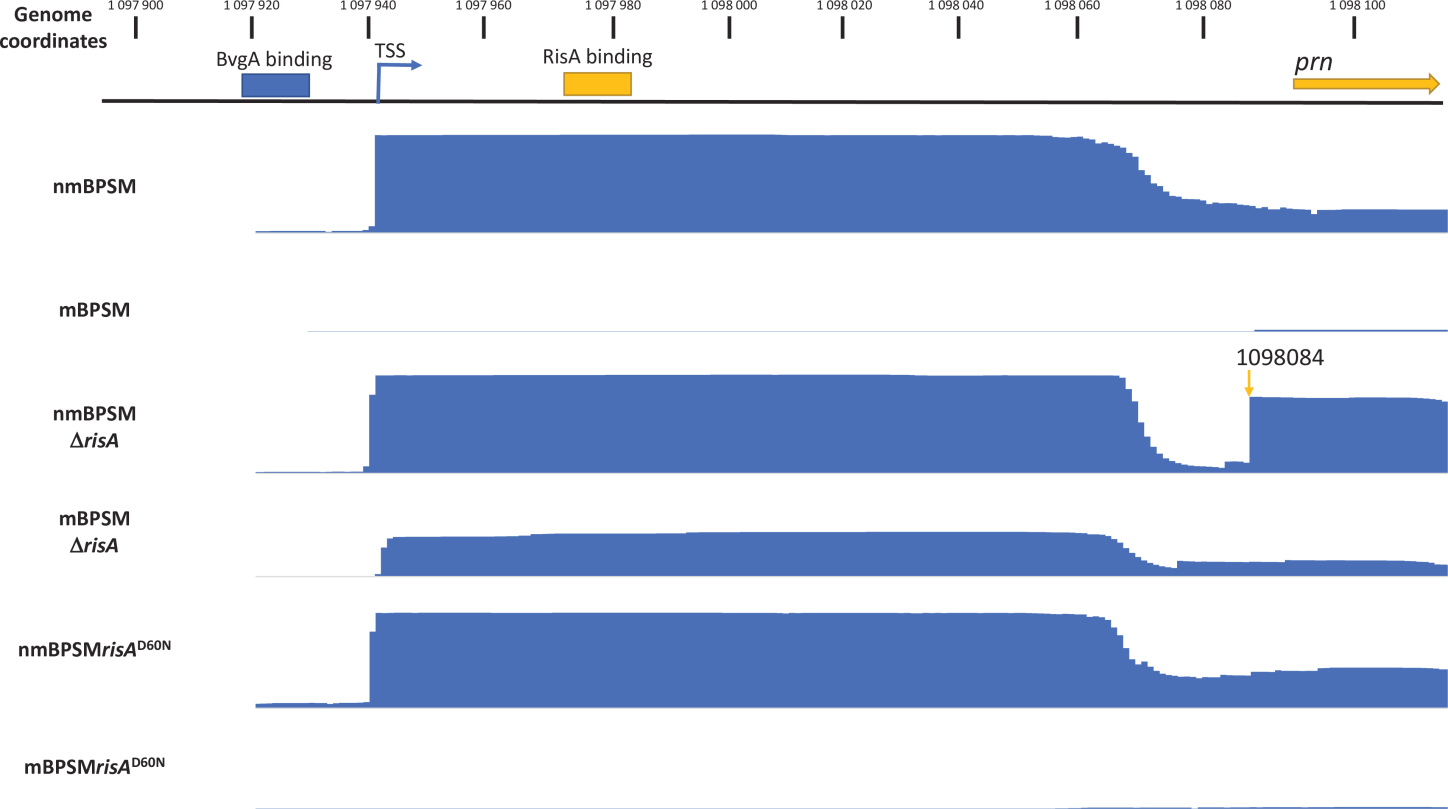
5′ RACE analysis of the *prn* promoter region. Schematic representation of the *B. pertussis prn* promoter region. The *prn* ORF start site is depicted by the yellow arrow, as annotated in the *B. pertussis* Tohama I BX470248 genome. Position of the TSS of *prn*, indicated by the blue arrow, was described by Kinnear et al. (11). Position of the BvgA-binding site, indicated by the blue box, was described in reference (2). Position of the RisA-binding site, indicated by the yellow box, is described in this study. Mapping of the RACE reads obtained with BPSM, BPSMΔ*risA,* and BPSM*risA*^D60N^ in modulating and non-modulating conditions are depicted in blue.

We were intrigued by the observation that for some genes, such as *prn*, expression was strongly downregulated in modulating conditions in BPSM and BPSM*risA*^D60N^, with Log_2_FC of −9.99 and −7.35, respectively, while the downregulation in modulating conditions was much less pronounced in BPSMΔ*risA*, with a Log_2_FC of only −3.71. In order to determine whether the decrease in regulation observed in BPSMΔ*risA* may be due to the use of a secondary promoter driving the expression of *prn*, we performed a RACE experiment to identify the 5′ end of the *prn* transcript in each condition. As shown in Fig. 6, only one transcriptional start site (TSS) was identified in nmBPSM, and nmBPSM*risA*^D60N^ at the same position as previously described (11). In modulating conditions, only very few reads were found in the *prn* promoter region of mBPSM and mBPSM*risA*^D60N^, in accordance with the low level of *prn* expression in these conditions. In contrast, in nmBPSMΔ*risA* and mBPSMΔ*risA,* many reads were found at the same TSS as in nmBPSM and nmBPSM*risA*^D60N^. These results indicate that the TSS used in non-modulating conditions to express *prn* is still active in modulating conditions but only when RisA is absent, arguing on a repressor function of RisA on this TSS regardless of its phosphorylation. This is consistent with the identification of a RisA-binding site downstream of the TSS. Interestingly, a new *prn* TSS was found in nmBPSMΔ*risA* at position 1,098,084. However, this TSS was not detected in mBPSMΔ*risA*, arguing that this TSS is regulated by both modulation and RisA.

### Identification of a RisA-motif consensus

**Fig 7:**
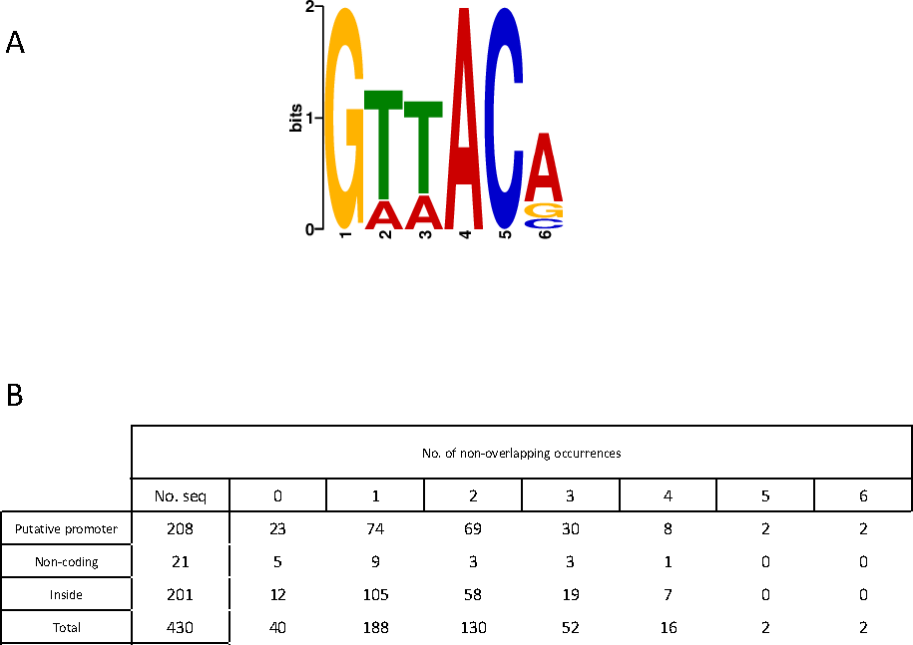
MEME analysis of the RisA ChIPseq peaks. (**A**) Motif found using the 430 RisA-binding sites. The number of sites contributing to the construction of the motif = 391, *E*-value 2.6 e^−106^, and 430 sequences used. (**B**) Number of RisA motif occurrences in the 430 RisA ChIPseq peaks according to the localization. Putative promoter regions are located between two ORFs orientated in opposite directions. Non-coding regions are not annotated in the *B. pertussis* Tohama I BX470248 genome. Inside means within annotated ORF of the *B. pertussis* Tohama I BX470248 genome. nt, nucleotides.

In an attempt to identify a RisA-binding consensus sequence based on the ChIPseq data, we used MEME ChIPseq software with the “Zero or one occurrence per sequence” or “Any number of repetitions” options to analyze the 430 RisA ChIPseq peaks. For each of them, we considered the mean of the center of the peak detected in all conditions and 100 bp on each side to define a 200-bp region, which allowed us to identify a 6-bp specific consensus GTTACA in 391 sequences, with an *E*-value of 2.6^e-106^ (Fig. 7A). The FIMO algorithm of the MEME Suite found this motif 691 times among the 391 regions of interest (Table S11). While 40 sequences do not contain the discovered motif, others contain up to six non-overlapping copies of this motif (Fig. 7B). A second analysis using only the regions not showing the detected motif did not allow us to identify another consensual motif. The distances between two consecutive motifs belonging to the same region range from 5 to 180 bp (data not shown). A further analysis using only the 208 regions corresponding to the RisA ChIPseq peaks in putative promoter regions and the 201 regions corresponding to the RisA ChIPseq peaks within ORFs did not allow us to identify a different motif. We also analyzed the regions corresponding to the 18 ChIPseq clusters where RisA binds in BPSM but not in BPSM*risA*^D60N^ to identify potential motives that would be specific for phosphorylated RisA, but we did not find a phosphorylation-specific RisA motif using these 18 200-bp regions. However, curiously, 8 of these 18 regions do not present the GTTACA motif.

## DISCUSSION

The two master regulatory systems of *B. pertussis* virulence are the two-component systems BvgA/S and RisA/K. While BvgA/S activates the expression of the *vag*s, including *bvgR* coding for a c-di-GMP phosphodiesterase, RisA/K activates the transcription of the *vrg*s. In previous studies, the BvgA regulon has been determined (2, 12). Here, we determined the complete RisA regulon using a combination of RNAseq and ChIPseq analyses and show that RisA may exert transcriptional repressor and activator functions. These regulatory functions may or may not require RisA phosphorylation or the presence of c-di-GMP, as shown by growing the organism under modulating conditions, in which c-di-GMP levels are predicted to be elevated due to the absence of BvgR. In this and previous studies (2, 12), *vag*s and *vrg*s were defined by the down- and upregulation, respectively, of their expression under modulating conditions, such as by culturing in the presence of MgSO_4_. We deliberately chose a high threshold to define *vag*s and *vrg*s (Log_2_FC of 3), because lower thresholds may increase the possibility of false assignments. Furthermore, by using this threshold, we could compare the fold changes seen here with those presented in earlier studies. Obviously, by using the high stringency of Log_2_FC, we may miss some *vag*s and *vrg*s.

In non-modulating conditions, the regulatory role of RisA on the transcriptome of *B. pertussis* is relatively limited. The 20 genes negatively regulated and the 49 genes positively regulated by RisA (Table S2) represent only 0.55% of the total transcript abundance of nmBPSM. In contrast, in modulating conditions, the 84 genes negatively regulated and the 77 genes positively regulated by RisA (Table S2) represent more than 11% of the total transcriptome of mBPSM, mainly because RisA is involved in the regulation of the *vrg*s. By comparing nmBPSM*∆risA* to nmBPSM, only seven *vrg*s presented a Log_2_FC < −3 indicating their regulation by RisA in non-modulating conditions. Using the less stringent Log_2_FC < −2 cutoff, 19 *vrg*s showed a weaker expression in nmBPSM*∆risA* compared to nmBPSM. However, for 21 *vrg*s, no modification of their expression was seen between nmBPSM*∆risA* and nmBPSM, suggesting that RisA did not regulate these genes in non-modulating conditions, while, with the exception of *bp1703-05, bp2089-90,* and *bp3858-61*, all the *vrg*s were regulated by RisA in modulating conditions. Thus, additional co-factors and/or partners that may be required to mediate the RisA regulation are probably missing in non-modulating conditions for the regulation of these genes.

Chen et al. (6) have shown that the production and phosphorylation of RisA do not differ between modulating and non-modulating conditions. Therefore, the modulation may act principally on the level of c-di-GMP determined by the regulation of *bvgR*. Some *vag*s showed differences in regulation by modulation between mBPSM*∆risA* and mBPSM; hence, some were no longer modulated in mBPSM*∆risA*, while for others regulation by modulation was reduced. These findings are consistent with previous microarray and qRT-PCR analyses (5).

By combining the RNAseq and ChIPseq data, we found that 10 *vag*s, including genes involved in adhesin production and maturation and the type III secretion system (*prn, brpL, bp1496,* and *fimB*), contain a RisA-binding site in their promoter region, suggesting that RisA is involved directly in the regulation of these genes. The *vag8* gene coding for the resistance to serum-killing protein also contains a RisA-binding site in its promoter region but was not regulated by RisA in the conditions tested here, thus perhaps requiring growth conditions different from those tested here for regulation by RisA. The operon encoding for the major adhesins FHA and fimbriae, *fhaB/fimABCD/fhaC* presented interesting differences in regulation by modulation between cistrons in mBPSM*∆risA*. While *fimBCD/fhaC* expression was strongly reduced by modulation in mBPSM*risA*^D60N^, *fhaB* and *fimA* transcription was less reduced. We found a RisA-binding site between *fimA* and *fimB*, suggesting that *fhaB/fimABCD/fhaC* are regulated by modulation, but a second level of regulation occurs for *fimBCD/fhaC* depending on RisA (Table S4). The three-gene operon *bp1703-05* was upregulated in both mBPSM and mBPSM*∆risA*, suggesting that it is not regulated by RisA. However, these three genes present a *vrg* profile of regulation, and a RisA-binding site was found in the promoter region of this operon upstream of *bp1705*, suggesting a complex regulation of this operon (Table S8).

While many RisA-binding sites are present in promoter regions of *vrg*s and *vag*s, most RisA-binding sites are present in promoter regions of genes not identified as *vrg*s or *vag*s by RNAseq analysis (164/208). It may be possible that these genes would be regulated by RisA in conditions other than those tested in this study. Alternatively, this RisA binding may be a residual of RisA regulation in other *Bordetella* species, such as *B. bronchiseptica*, since *B. pertussis* has likely evolved from a *B. bronchiseptica*-like ancestor (13).

The RNAseq analysis also identified 189 regulated regions not related to previously annotated ORFs, among them nine are not 3′ or 5′ UTR and may be putative RisA-regulated sRNA. These regions could not be identified in the previous microarray study (5), but some of them have been predicted in a previous study (14). Transcript_301, Transcript_356, *rgtA,* and *vrgX* were already described as sRNA (2, 10, 15). Novel_transcript_1 was identified as putative sRNA regulated by modulation and shown previously to present a BvgA-binding site (2). Here, we identified four additional putative sRNA. The expression of Novel_transcript_2 was increased in mBPSM, mBPSMΔ*risA,* and mBPSM*risA*^D60N^, suggesting regulation by modulation but not by RisA. However, the ChIPseq data showed a strong RisA binding in that region, suggesting that RisA may be involved in the regulation of this region, but this was not apparent in RNAseq in the tested conditions. Novel_transcript_1 and Novel_transcript_2 are transcribed in the same orientation and very close to the 5′ and their adjacent ORFs. Further experiments will be required to exclude the possibility that these novel RNAs might be UTRs of *bp0258* and *bp0278*, respectively. Novel_transcript_3, Novel_transcript_4, and Novel_transcript_5 were found by RNAseq to be regulated in modulated and non-modulated BPSM*∆risA* and BPSM*risA*^D60N^ and present a RisA-binding site, suggesting that their expression is directly regulated by RisA.

Several genes are regulated by RisA and/or modulation code for putative regulators and may hence regulate other genes by cascade regulations. The *bp1022* gene, which contains a RisA-binding site in its promoter, was shown to regulate the chemotaxis and flagellar operons (5). We have reported previously that *bp1022* also presents a BvgA-binding site (2). BvgA and RisA may thus conjointly regulate the chemotaxis and flagellar operons through the regulation by BP1022.

Two two-component-system genes were also found to be regulated by RisA based on the RNAseq data and present a RisA-binding site in their promoter regions. One of them, *bp1496*, was also shown to be regulated by BvgA (2). RisA also regulates the expression of *brpL*, coding for a sigma factor that regulates the expression of the type III secretion system operons, which is also regulated by BvgA and contains a BvgA-binding site in its promoter region (2). Here, we show that it also contains a RisA-binding site in its promoter region, suggesting a double BvgA and RisA regulation.

Of the 27 genes coding for putative regulators presenting a RisA-binding site, 5 genes, *bp1091*, *bp1496*, *bvgR,* and *brpL,* were found to be regulated according to our RNAseq data, suggesting a possible RisA-cascade regulation in conditions other than those tested here (Table S10). *risA* itself also contains a RisA-binding site in its promoter region. However, in our tested conditions, we have no evidence for RisA autoregulation. In addition, no RisA-binding site was found in the promoter region of the *ompR/risK* operon coding for the cognate RisA kinase (2, 6).

Many RisA-binding sites were detected within ORFs. Although the function of such binding is yet unknown, many ChIPseq studies with other bacteria have demonstrated that transcription factors may bind to sites within ORFs (2, 16–20). Fifty of these intra-ORF RisA-binding sites were found within the first 10% of the corresponding ORF (Fig. 5). As miss annotations of translational initiation codons of the Tohama I BX470248 genome annotation have been shown to occur (21), it is possible that some of these RisA-binding sites may in fact be located upstream of the actual translational start codon and would therefore be located within the promoter region. For at least four of them (*bp2216, bp1709, bp3300,* and *bp3689*) this is likely the case, based on ortholog annotation in other genomes. Furthermore, 28 RisA-binding sites were detected in the last 10% of ORFs, of which three correspond to overlapping genes (*bp1136*, *bp2438,* and *bp3766*) and eight to genes with less than 50 bp between the two ORFs transcribed in the same orientation (including *bp1875*, just upstream of *bvgR*), suggesting that they may correspond to binding sites in the promoter regions of the downstream ORFs.

A comparison of the RisA ChIPseq data described here with the previously published BvgA ChIPseq data (2) allowed us to identify 48 regions of the *B. pertussis* genome that contain both a BvgA- and a RisA-binding sites (Table S12). Six of them correspond to ORFs of *vag*s or *vrg*s identified by RNAseq, while 39 were not identified as such. This represents one-fourth of the previously identified BvgA-binding sites and suggests that RisA and BvgA may function jointly to regulate the expression of many genes involved in *B. pertussis* virulence and metabolism.

We have taken a closer look at the *prn* promoter region and found that it contains both a BvgA- and a RisA-binding site. The TSS of *prn* was previously identified 149 bp upstream of the *prn* translational initiation codon in the *B. pertussis* Tohama I BX470248 genome (11). By RACE analysis, we confirmed the previously described *prn* TSS. Using DNase I protection studies Kinnear et al. (11) found two regions of BvgA binding at positions −94 to −52 and −51 to +22 relative to the TSS. By ChIPseq analysis, we have also previously shown that *prn* contains a BvgA-binding site between positions −183 and +155 relative to this TSS with a center of the peak at position −14 (2). We found here that *prn* also contains a RisA-binding site at position +34 relative to this TSS. The *prn* TSS thus lies between an upstream BvgA-binding site and a downstream RisA-binding site (Fig. 6). Thus, while BvgA may activate *prn* expression in non-modulating conditions, RisA may repress its expression in modulating and non-modulating conditions. Repression of gene expression by binding of regulators downstream of the TSS was already demonstrated for BvgA binding downstream of the TSS of the intimin gene *bipA* (22). Interestingly, a second *prn* TSS was found by RACE analysis in nmBPSMΔ*risA*. This TSS is located downstream of the RisA-binding site and 142 bp downstream of the first TSS. The use of this TSS is regulated by modulation, thus likely by BvgA, and may correspond to the BvgA-binding site described by Kinnear et al. between −51 and +22 (11) relative to the primary *prn* TSS and to the extended BvgA ChIPseq peak downstream of the primary TSS (2).

A MEME ChIPseq analysis of the regions containing a RisA-binding site allowed us to identify the 6 bp specific consensus GTTACA. Crónín et al. (23) have proposed a putative consensus RisA-binding site based on an alignment of sequences from the *vrg6*, *vrg18*, *vrg24,* and *vrg73* promoter regions. They proposed a 7-bp consensus sequence TTTAC/AAT. One of the 6-bp RisA consensus found by MEME overlaps one of the 7-bp predicted RisA sequence found on the *vrg6* promoter of *B. pertussis* by Cronin et al. (23), arguing that these two consensus sequences are very close. The strength of the RisA consensus discovered by ChIPseq analysis is that it is based on 391 binding sites containing 691 consensual motifs.

### Conclusions

**Fig 8:**
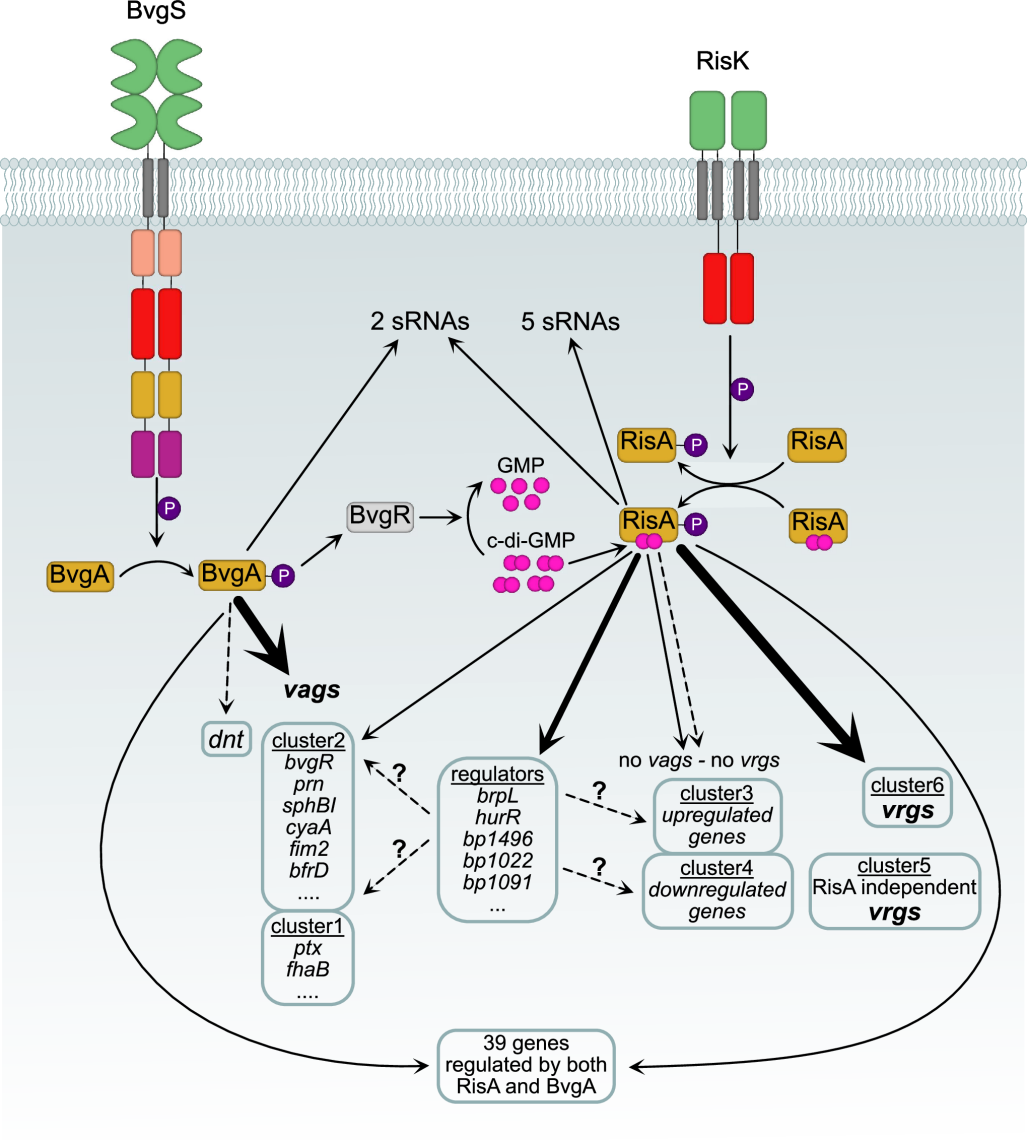
Proposed model of gene regulation by BvgA and RisA in *B. pertussis*. The kinases BvgS and RisK phosphorylate BvgA and RisA, respectively. In avirulent conditions, *bvgR* is not expressed, and therefore c-di-GMP levels are elevated and serve as a co-factor of RisA, which regulates the expression of several clusters of genes directly by binding to the corresponding promoter region, or indirectly, via a regulation cascade. Solid lines represent direct regulation, as demonstrated by the ChIPseq results, and dashed lines represent indirect regulation, as demonstrated by the ChIPseq and RNAseq results.

In summary, by using a combined RNAseq and ChIPseq analysis, we have determined here the RisA regulon of *B. pertussis* Tohama I and suggest some RisA regulation cascades, as several RisA-regulated genes containing a RisA-binding site code for putative regulatory functions, including putative small non-coding RNAs, whereas some RisA-regulated genes did not contain a RisA-binding site and thus be indirectly regulated by RisA. The study revealed a number of surprises. Although, as expected, RisA-binding sites were identified in the promoter regions of *vrg*s, as determined by RNAseq analysis, some were also detected in promoter regions of *vag*s, suggesting a previously not appreciated complex regulation mechanism of these *vag*s. Moreover, in the absence of RisA, the expression of some of the *vag*s was no longer affected by modulation, further illustrating the unexpected fine-tuning role of RisA on the expression of some *vag*s. In addition, most RisA-binding sites were identified within ORFs and in promoter regions of genes not regulated by modulation in the conditions used here, suggesting that RisA may play a regulatory role in many more conditions than the classical *in vitro* growth conditions routinely used to study the *B. pertussis* virulence genes. Of note, with some exceptions, e.g., for certain *vags*, the effect of the D^60^N mutation in RisA is very similar to the effect of the *risA* deletion. In addition, RisA regulation is similar in modulating and non-modulating conditions, albeit somewhat weaker in the latter than in the former conditions. Collectively, the observations presented here, combined with previous studies, led us to propose a model of the BvgA/S-RisA/K interplay depicted in Fig. 8. Phosphorylated BvgA activates directly or indirectly the transcription of *vag*s, including *bvgR*, coding for c-di-GMP phosphodiesterase, which results in a decrease in intracellular c-di-GMP levels, a co-factor of RisA. In avirulent conditions, RisA, phosphorylated by the kinase RisK, and in the presence of c-di-GMP, directly or indirectly activates the transcription of several clusters of genes, including several genes coding for transcriptional regulators, suggesting a regulation cascade. Surprisingly, 39 genes are regulated by both BvgA and RisA.

## MATERIALS AND METHODS

### Construction of *B*. *pertussis* mutant strains

The *B. pertussis* strains used in this study were derived from the Tohama I derivative BPSM (9) and BPSM∆*risA* (5). BPSM*risA*^D60N^ was obtained as follows. A 1,300-bp fragment containing the *risA*^D60N^ version of *risA* was PCR amplified using oligonucleotide pairs 5′- TATAgatcctgggcaacaggcgttctgca-3′ and 5′- TATAAAGCTTTTCGTTGGTGGCCAGGTCCA-3′ and the *B. pertussis* QC3296 genomic DNA (5). The resulting PCR product was digested by *Bam*HI and *Hin*dIII and introduced into pSS1129 (24), yielding pSS1129*risA*^D60N^. This construct was used for allelic exchange, as described in reference (2), in BPSM∆*risA*, yielding BPSM*risA*^D60N^ that produces a phosphoablative form of RisA. The recombinant plasmids were introduced into *B. pertussis* by conjugation via *Escherichia coli* SM10 (25).

### RNA extractions

*B. pertussis* strains were grown on BG agar plates for 2 days at 37°C and then cultured in Stainer Scholte (SS) liquid medium supplemented, when indicated, with 50 mM MgSO_4_ at 37°C under agitation. The bacterial cultures were stopped at the mid-exponential phase (OD_600_ = 1.5–2) by adding 2 mL of 5:95 phenol:ethanol (vol/vol) to 8 mL of bacterial suspension. Bacteria were pelleted, and total RNA was extracted using TriReagent (Invitrogen) following the manufacturer’s instructions. Genomic DNA was removed by DNase I treatment (Sigma-Aldrich).

### Illumina RNA sequencing

RNA-seq was performed on two independent cultures for each condition. For each RNAseq sample, DNA-depleted total RNA was treated with the Ribo-Zero rRNA Removal Kit (Illumina) following the manufacturer’s recommendations. The rRNA-depleted RNA was then used to build the Illumina library using the TruSeq RNA Library Preparation Kit, followed by sequencing on an Illumina NextSeq 500 benchtop sequencer on SR150 high output run mode. The RNAseq data of each sample were analyzed using Rockhopper v2.0.3 and SPARTA with the default parameters to calculate the RPKM and *P* values for each coding sequence using the *B. pertussis* Tohama I BX470248 genome annotation (26).

### Production of recombinant RisA and anti-RisA antibodies

To produce recombinant RisA, *risA* was amplified by PCR using the 5′-TATACATATGAACACGCAAAACACCACTCCT-3′ and 5′-TATAGGATCCTCAACTGCCGCCATCCGGAACGAA-3′ primers and the BPSM genomic DNA as a template. The amplicon was introduced into pET15b (Novagen), yielding pET15bRisA, which was used to transform BL21(DE3) for 6-HisTag-RisA protein production. 6-HisTag-RisA was purified using the HiTrap Amersham column. Guinea pigs were immunized by Eurogentec with purified RisA. Immune sera were harvested after 28 days. Non-specific antibodies were removed from the immune sera by depletion on nitrocellulose membrane charged with a BPSMΔ*risA* total lysate.

### ChIP analysis

The *B. pertussis* ChIP protocol was adapted from Solans et al. (27). *B. pertussis* strains were grown on BG agar plates for 2 days at 37°C and then cultured in modified SS liquid medium supplemented, when indicated, with 50 mM MgSO_4_ at 37°C under agitation. The bacterial cultures were stopped at the mid-exponential phase (OD_600_ = 1.5–2) by adding 1% final concentration of formaldehyde and incubated for 30 min at 37°C with gentle shaking. One hundred twenty-five millimolar glycine was added to saturate the crosslinking reaction and incubation was continued for 30 min at 37°C with gentle shaking. The bacterial suspension was then centrifuged at 5,000 × *g* for 10 min and washed twice with PBS. Cell pellets were resuspended in Immunoprecipitation buffer + Antiprotease mini (Roche). Bacteria were lysed by sonication with a refrigerated Diagenode Bioruptor at 4°C with specific TPX Diagenode tubes. Cell debris was removed by centrifugation (15 min, 4,000 rpm, 4°C). To check the DNA fragmentation before immunoprecipitation (IP), a sample of DNA fragments was heated for 6 h at 65°C, treated with RNase A and Proteinase K, and precipitated. The fragment size distribution was checked on BioAnalyser. IP was performed with anti-RisA anti-serum and 150- to 600-bp *B. pertussis* DNA fragments. The samples were incubated at 4°C on a spinning wheel for 16 h. Protein G Magnetic Dynabeads (Invitrogen) were added to the IP samples and incubated for 4 h at 4°C on the spinning wheel. Beads were separated from the lysate by using a magnet and washed with IP buffer as described (27). Beads were finally resuspended in TE buffer and incubated for 6 h at 65°C. The supernatant was then treated with RNase A and Proteinase K, and the DNA fragments were extracted with phenol-chloroform and precipitated with isopropanol. The ChIP procedure was done on two independent cultures on each strain and on one culture of BPSM without anti-RisA anti-serum as ChIP negative control.

### Illumina ChIPseq

The DNA fragments isolated by ChIP were used to build the Illumina library using the Illumina TruSeq ChIP Library Preparation Kit, followed by sequencing on an Illumina NextSeq 500 benchtop sequencer on SR150 high output run mode. The ChIPseq data of each sample were analyzed using the ChIP-Seq Analysis module of CLC genomics v11.0 using the default parameters, and the *B. pertussis* Tohama I BX470248 genome annotation to map reads, do the peak calling, and calculate peak shape scores. To avoid false positives and to increase clarity, ChIPseq peaks were considered informative if the CLC genomic peak shape score was >5. The mapped read depth was calculated on ChIPseq CLC genomics output bam files using the SAMtools depth module of SAMtools (28).

### RACE analysis

For each RACE sample, total RNA, extracted as described above, was treated with GeneRacer kit (Invitrogen) according to the manufacturer’s instructions and using the *prn* RACE primer 5′-CGCTCACCGGTCTTGACGAT-3′. The obtained cDNA was then used for the nested PCR using *prn* Nested primer 5′-CTTGACAATGCGTGACAGAGACA-3′ and the kit-included GeneRacer Nested primer. The nested PCR amplicon was then used to build the Illumina library using the Illumina TruSeq ChIP Library Preparation Kit, followed by sequencing on an Illumina NextSeq 500 benchtop sequencer on SR150 low output run mode. The mapped read depth was calculated on RACE CLC genomics output bam files using the SAMtools depth module of SAMtools (28)

## Data Availability

RNAseq data have been deposited at NCBI Sequence Read Archive (SRA) under accession PRJNA474836 and PRJNA756173, ChIPseq data were deposited at SRA under accession PRJNA756196, and RACE data were deposited at SRA under accession PRJNA770881.

## SUPPLEMENTAL MATERIAL

Supplemental Figures msystems.00951-23-s0001.pdf Figures S1 to S15.

Supplemental Tables msystems.00951-23-s0002.xlsx Tables S1 to S12.

ASM does not own the copyrights to Supplemental Material that may be linked to, or accessed through, an article. The authors have granted ASM a non-exclusive, world-wide license to publish the Supplemental Material files. Please contact the corresponding author directly for reuse.

## References

[1] Chen Q, Stibitz S. 2019. The BvgASR virulence regulon of Bordetella pertussis. Curr Opin Microbiol 47:74–81. doi:10.1016/j.mib.2019.01.002 30870653

[2] Coutte L, Antoine R, Slupek S, Solans L, Derop J, Bonnefond A, Hot D, Locht C. 2020. Combined RNAseq and ChIPseq analyses of the BvgA virulence regulator of Bordetella pertussis. mSystems 5:5. doi:10.1128/mSystems.00208-20 PMC7253368 32430408

[3] Dupré E, Herrou J, Lensink MF, Wintjens R, Vagin A, Lebedev A, Crosson S, Villeret V, Locht C, Antoine R, Jacob-Dubuisson F. 2015. Virulence regulation with venus flytrap domains: structure and function of the periplasmic moiety of the sensor-kinase BvgS. PLoS Pathog 11:e1004700. doi:10.1371/journal.ppat.1004700 25738876 PMC4352136

[4] Lacey BW. 1960. Antigenic modulation of Bordetella pertussis. J Hyg (Lond) 58:57–93. doi:10.1017/s0022172400038134 14413273 PMC2134314

[5] Coutte L, Huot L, Antoine R, Slupek S, Merkel TJ, Chen Q, Stibitz S, Hot D, Locht C. 2016. The multifaceted RisA regulon of Bordetella pertussis. Sci Rep 6:32774. doi:10.1038/srep32774 27620673 PMC5020355

[6] Chen Q, Ng V, Warfel JM, Merkel TJ, Stibitz S. 2017. Activation of Bvg-repressed genes in Bordetella pertussis by RisA requires cross talk from noncooperonic histidine kinase RisK. J Bacteriol 199:199. doi:10.1128/JB.00475-17 PMC5648863 28827216

[7] Merkel TJ, Barros C, Stibitz S. 1998. Characterization of the bvgR locus of Bordetella pertussis. J Bacteriol 180:1682–1690. doi:10.1128/JB.180.7.1682-1690.1998 9537363 PMC107078

[8] Trainor EA, Nicholson TL, Merkel TJ. 2015. Bordetella pertussis transmission. Pathog Dis 73:ftv068. doi:10.1093/femspd/ftv068 26374235 PMC4626651

[9] Menozzi FD, Mutombo R, Renauld G, Gantiez C, Hannah JH, Leininger E, Brennan MJ, Locht C. 1994. Heparin-inhibitable lectin activity of the filamentous hemagglutinin adhesin of Bordetella pertussis. Infect Immun 62:769–778. doi:10.1128/iai.62.3.769-778.1994 8112848 PMC186182

[10] Chen Q, Lee G, Craig C, Ng V, Carlson PE, Hinton DM, Stibitz S. 2018. A novel Bvg-repressed promoter causes vrg-like transcription of fim3 but does not result in the production of serotype 3 fimbriae in Bvg^-^ mode Bordetella pertussis. J Bacteriol 200:e00175-18. doi:10.1128/JB.00175-18 30061354 PMC6153668

[11] Kinnear SM, Boucher PE, Stibitz S, Carbonetti NH. 1999. Analysis of BvgA activation of the pertactin gene promoter in Bordetella pertussis. J Bacteriol 181:5234–5241. doi:10.1128/JB.181.17.5234-5241.1999 10464192 PMC94027

[12] Moon K, Bonocora RP, Kim DD, Chen Q, Wade JT, Stibitz S, Hinton DM. 2017. The BvgAS regulon of Bordetella pertussis. mBio 8. doi: 10.1128/mBio.01526-17 10.1128/mBio.01526-17 PMC5635692 29018122

[13] Diavatopoulos DA, Cummings CA, Schouls LM, Brinig MM, Relman DA, Mooi FR. 2005. Bordetella pertussis, the causative agent of whooping cough, evolved from a distinct, human-associated lineage of B. bronchiseptica. PLoS Pathog 1:e45. doi:10.1371/journal.ppat.0010045 16389302 PMC1323478

[14] Amman F, D’Halluin A, Antoine R, Huot L, Bibova I, Keidel K, Slupek S, Bouquet P, Coutte L, Caboche S, Locht C, Vecerek B, Hot D. 2018. Primary transcriptome analysis reveals importance of IS elements for the shaping of the transcriptional landscape of Bordetella pertussis. RNA Biol 15:967–975. doi:10.1080/15476286.2018.1462655 29683387 PMC6161684

[15] Keidel K, Amman F, Bibova I, Drzmisek J, Benes V, Hot D, Vecerek B. 2018. Signal transduction-dependent small regulatory RNA is involved in glutamate metabolism of the human pathogen Bordetella pertussis. RNA 24:1530–1541. doi:10.1261/rna.067306.118 30097543 PMC6191719

[16] Bonocora RP, Fitzgerald DM, Stringer AM, Wade JT. 2013. Non-canonical protein-DNA interactions identified by ChIP are not artifacts. BMC Genomics 14:254. doi:10.1186/1471-2164-14-254 23586855 PMC3738151

[17] Shimada T, Ishihama A, Busby SJW, Grainger DC. 2008. The Escherichia coli RutR transcription factor binds at targets within genes as well as intergenic regions. Nucleic Acids Res 36:3950–3955. doi:10.1093/nar/gkn339 18515344 PMC2475637

[18] Wade JT, Struhl K, Busby SJW, Grainger DC. 2007. Genomic analysis of protein-DNA interactions in bacteria: insights into transcription and chromosome organization. Mol Microbiol 65:21–26. doi:10.1111/j.1365-2958.2007.05781.x 17581117

[19] Galagan J, Lyubetskaya A, Gomes A. 2013. ChIP-Seq and the complexity of bacterial transcriptional regulation. Curr Top Microbiol Immunol 363:43–68. doi:10.1007/82_2012_257 22983621

[20] Fitzgerald DM, Stringer AM, Smith C, Lapierre P, Wade JT. 2023. Genome-wide mapping of the Escherichia coli PhoB regulon reveals many transcriptionally inert, intragenic binding sites. mBio 14:e0253522. doi:10.1128/mbio.02535-22 37067422 PMC10294691

[21] Rivera-Millot A, Lesne E, Solans L, Coutte L, Bertrand-Michel J, Froguel P, Dhennin V, Hot D, Locht C, Antoine R, Jacob-Dubuisson F. 2017. Characterization of a Bvg-regulated fatty acid methyl-transferase in Bordetella pertussis. PLoS One 12:e0176396. doi:10.1371/journal.pone.0176396 28493897 PMC5426589

[22] Williams CL, Boucher PE, Stibitz S, Cotter PA. 2005. BvgA functions as both an activator and a repressor to control Bvg phase expression of bipA in Bordetella pertussis. Mol Microbiol 56:175–188. doi:10.1111/j.1365-2958.2004.04526.x 15773988

[23] Cróinín TO, Grippe VK, Merkel TJ. 2005. Activation of the vrg6 promoter of Bordetella pertussis by RisA. J Bacteriol 187:1648–1658. doi:10.1128/JB.187.5.1648-1658.2005 15716435 PMC1063992

[24] Stibitz S, Black W, Falkow S. 1986. The construction of a cloning vector designed for gene replacement in Bordetella pertussis. Gene 50:133–140. doi:10.1016/0378-1119(86)90318-5 2884169

[25] Simon R, Priefer U, Pühler A. 1983. A broad host range mobilization system for in vivo genetic engineering: transposon mutagenesis in Gram negative bacteria. Nat Biotechnol 1:784–791. doi:10.1038/nbt1183-784

[26] McClure R, Balasubramanian D, Sun Y, Bobrovskyy M, Sumby P, Genco CA, Vanderpool CK, Tjaden B. 2013. Computational analysis of bacterial RNA-Seq data. Nucleic Acids Res 41:e140. doi:10.1093/nar/gkt444 23716638 PMC3737546

[27] Solans L, Gonzalo-Asensio J, Sala C, Benjak A, Uplekar S, Rougemont J, Guilhot C, Malaga W, Martín C, Cole ST. 2014. The PhoP-dependent ncRNA Mcr7 modulates the TAT secretion system in Mycobacterium tuberculosis. PLoS Pathog 10:e1004183. doi:10.1371/journal.ppat.1004183 24874799 PMC4038636

[28] Li H. 2011. A statistical framework for SNP calling, mutation discovery, association mapping and population genetical parameter estimation from sequencing data. Bioinformatics 27:2987–2993. doi:10.1093/bioinformatics/btr509 21903627 PMC3198575

