# Supplementary material for: Combined transcriptomic and ChIPseq analyses of the Bordetella pertussis RisA regulon
Source: mSystems. 2024 Mar 12;9(4):e00951-23. doi: 10.1128/msystems.00951-23 (PMC11019879; doi:10.1128/msystems.00951-23)
Supplement: Supplemental Figures — Figures S1 to S15. [file msystems.00951-23-s0001.pdf]

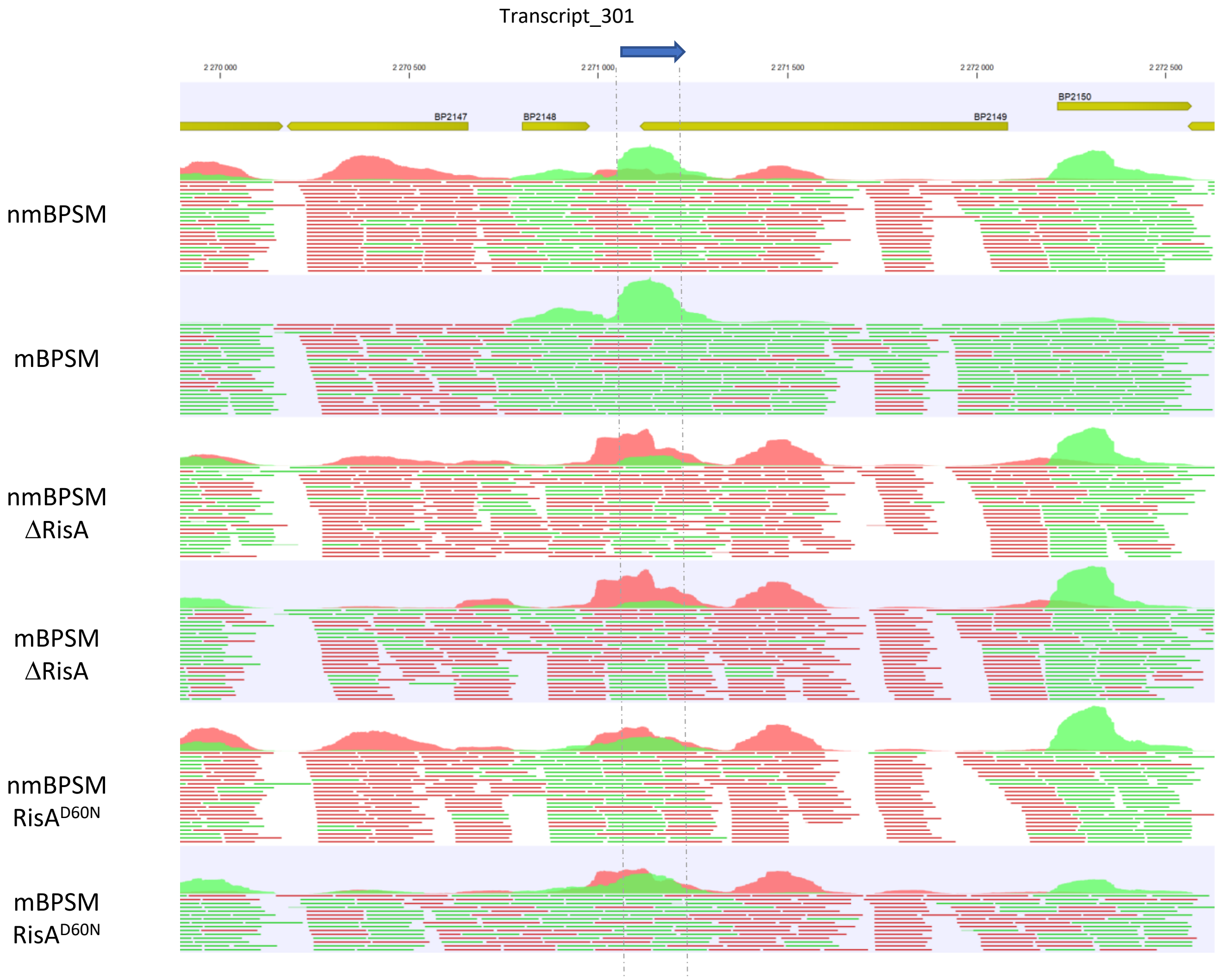

Figure S1. Localization of Transcript_301 a putative sRNA regulated by RisA. Schematic representation of the genetic environment of the sRNA with the adjacent ORFs in the *B. pertussis* Tohama I BX470248 genome. The respective transcripts and the orientation of their expression are indicated by the blue arrows, while the surrounding ORFs and their orientation are indicated by the yellow arrows. bp numbers correspond to the genome coordinates as annotated in the *B. pertussis* Tohama I BX470248 genome. Single reads mapping in the forward direction are in green. Single reads mapping in the reverse direction are red

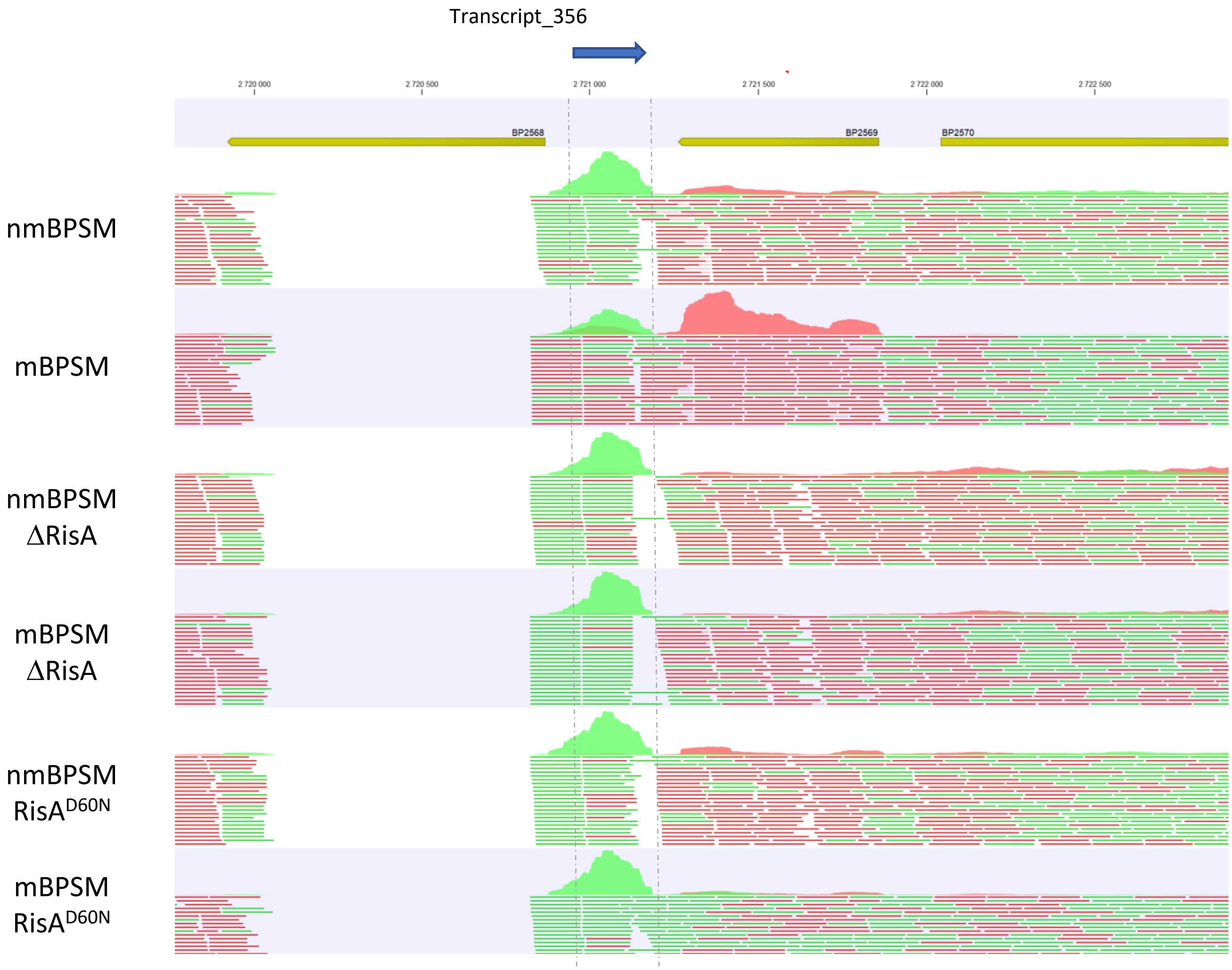

Figure S2. Localization of Transcript_356 a putative sRNA regulated by RisA. Schematic representation of the genetic environment of the sRNA with the adjacent ORFs in the *B. pertussis* Tohama I BX470248 genome. The respective transcripts and the orientation of their expression are indicated by the blue arrows, while the surrounding ORFs and their orientation are indicated by the yellow arrows. bp numbers correspond to the genome coordinates as annotated in the *B. pertussis* Tohama I BX470248 genome. Single reads mapping in the forward direction are in green. Single reads mapping in the reverse direction are red

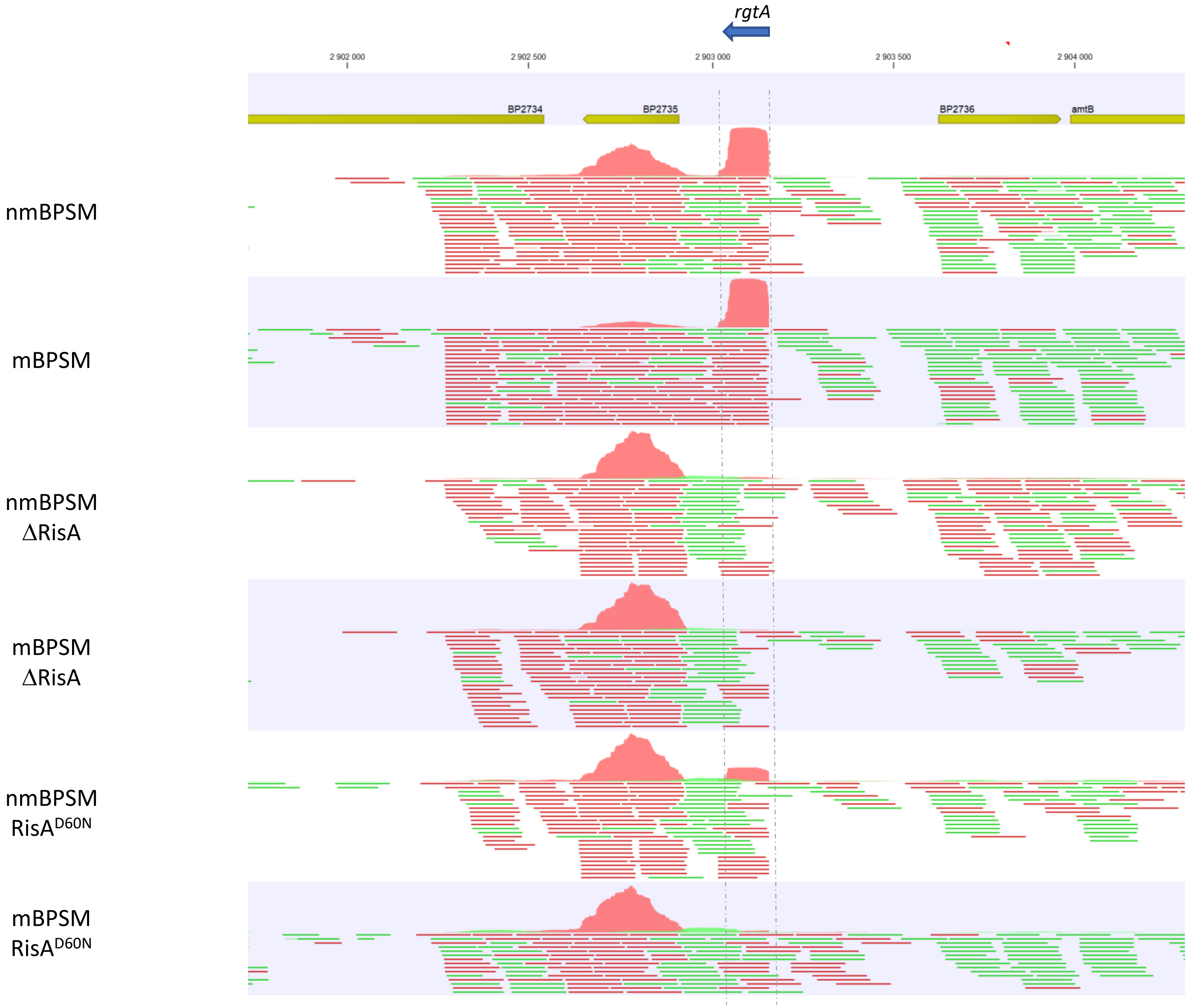

Figure S3. Localization of rgtA a sRNA regulated by RisA. Schematic representation of the genetic environment of the sRNA with the adjacent ORFs in the *B. pertussis* Tohama I BX470248 genome. The respective transcripts and the orientation of their expression are indicated by the blue arrows, while the surrounding ORFs and their orientation are indicated by the yellow arrows. bp numbers correspond to the genome coordinates as annotated in the *B. pertussis* Tohama I BX470248 genome. Single reads mapping in the forward direction are in green. Single reads mapping in the reverse direction are red

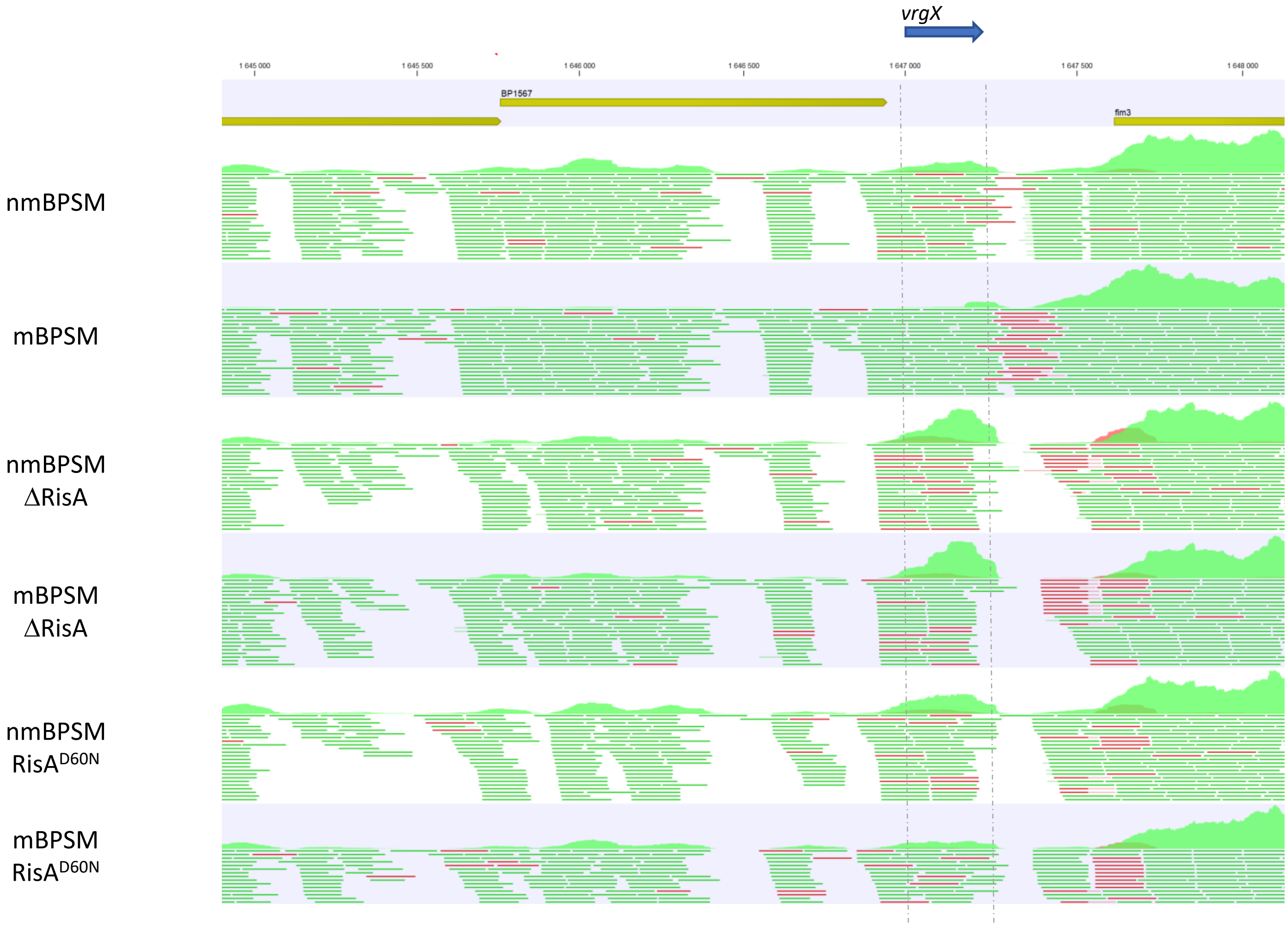

Figure S4. Localization of vrgX a sRNA regulated by RisA. Schematic representation of the genetic environment of the sRNA with the adjacent ORFs in the *B. pertussis* Tohama I BX470248 genome. The respective transcripts and the orientation of their expression are indicated by the blue arrows, while the surrounding ORFs and their orientation are indicated by the yellow arrows. bp numbers correspond to the genome coordinates as annotated in the *B. pertussis* Tohama I BX470248 genome. Single reads mapping in the forward direction are in green. Single reads mapping in the reverse direction are red.

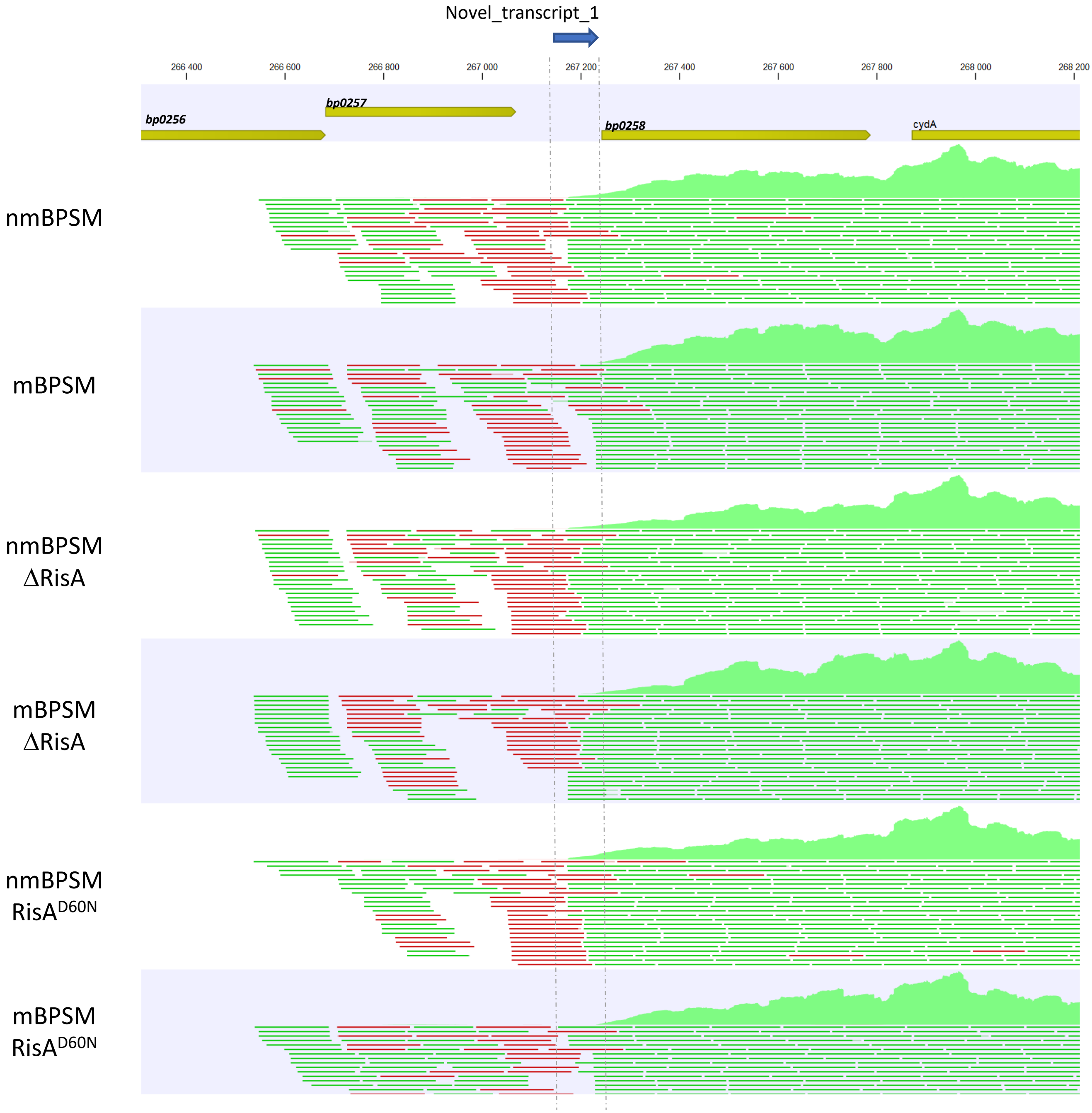

Figure S5. Localization of Novel_transcript_1 a putative sRNA regulated by RisA. Schematic representation of the genetic environment of the sRNA with the adjacent ORFs in the *B. pertussis* Tohama I BX470248 genome. The respective transcripts and the orientation of their expression are indicated by the blue arrows, while the surrounding ORFs and their orientation are indicated by the yellow arrows. bp numbers correspond to the genome coordinates as annotated in the *B. pertussis* Tohama I BX470248 genome. Single reads mapping in the forward direction are in green. Single reads mapping in the reverse direction are red

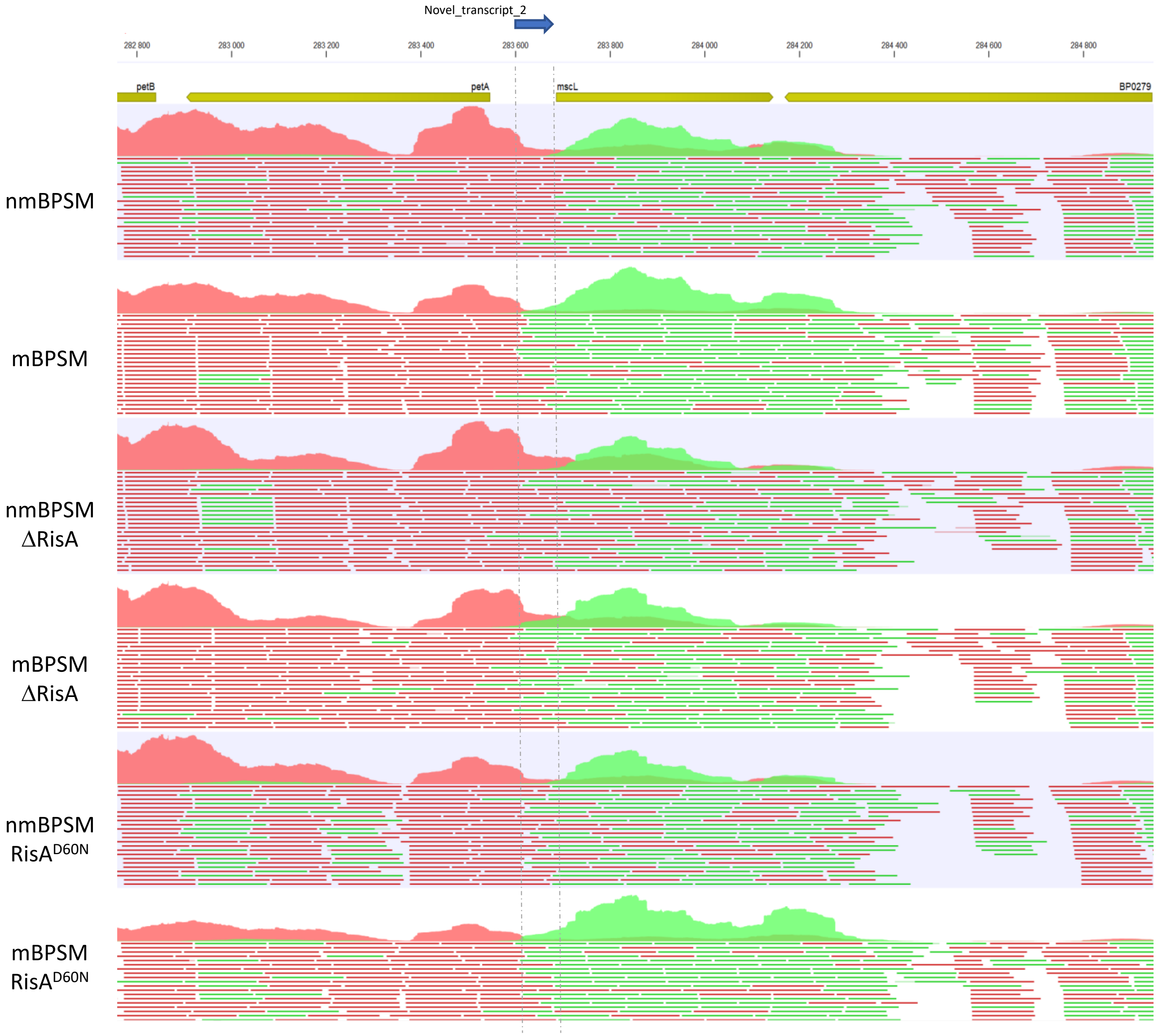

Figure S6. Localization of Novel_transcript_2 a putative sRNA regulated by RisA. Schematic representation of the genetic environment of the sRNA with the adjacent ORFs in the *B. pertussis* Tohama I BX470248 genome. The respective transcripts and the orientation of their expression are indicated by the blue arrows, while the surrounding ORFs and their orientation are indicated by the yellow arrows. bp numbers correspond to the genome coordinates as annotated in the *B. pertussis* Tohama I BX470248 genome. Single reads mapping in the forward direction are in green. Single reads mapping in the reverse direction are red

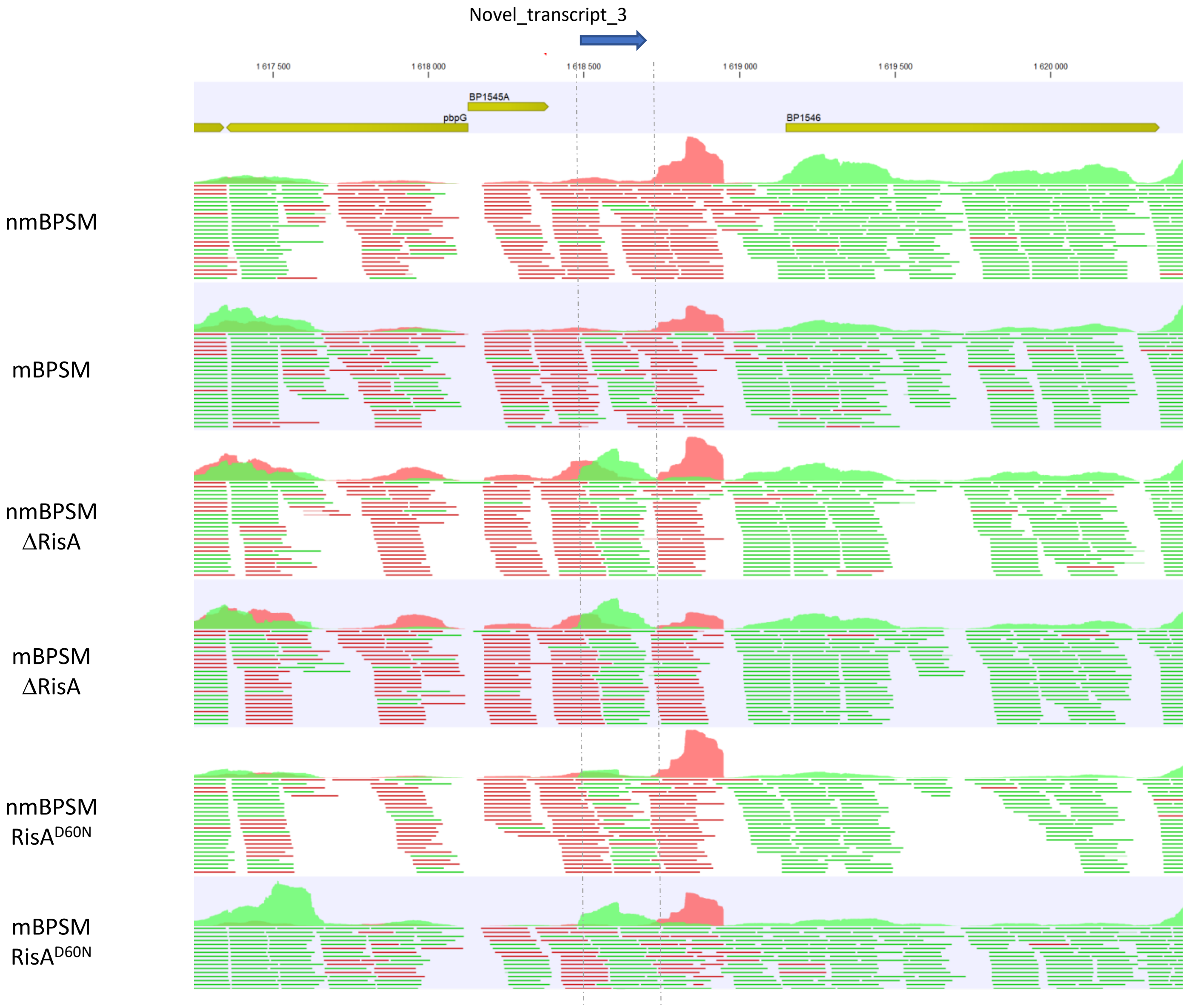

Figure S7. Localization of Novel_transcript_3 a putative sRNA regulated by RisA. Schematic representation of the genetic environment of the sRNA with the adjacent ORFs in the *B. pertussis* Tohama I BX470248 genome. The respective transcripts and the orientation of their expression are indicated by the blue arrows, while the surrounding ORFs and their orientation are indicated by the yellow arrows. bp numbers correspond to the genome coordinates as annotated in the *B. pertussis* Tohama I BX470248 genome. Single reads mapping in the forward direction are in green. Single reads mapping in the reverse direction are red

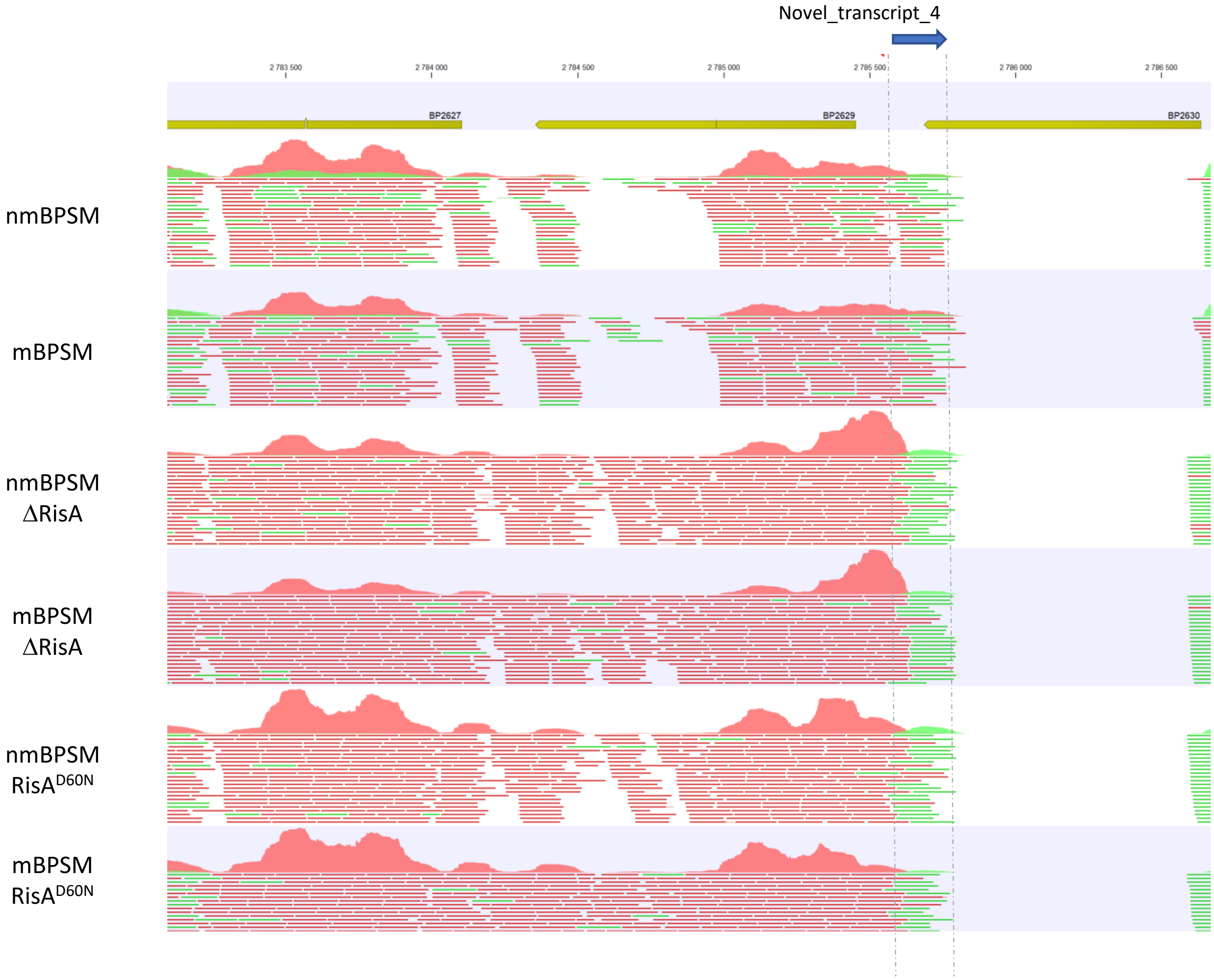

Figure S8. Localization of Novel_transcript_4 a putative sRNA regulated by RisA. Schematic representation of the genetic environment of the sRNA with the adjacent ORFs in the *B. pertussis* Tohama I BX470248 genome. The respective transcripts and the orientation of their expression are indicated by the blue arrows, while the surrounding ORFs and their orientation are indicated by the yellow arrows. bp numbers correspond to the genome coordinates as annotated in the *B. pertussis* Tohama I BX470248 genome. Single reads mapping in the forward direction are in green. Single reads mapping in the reverse direction are red. Single reads mapping in *bp3630* are not represented as *bp2630* codes for a transposase for IS481 element and reads from IS were excluded during the RNAseq mapping.

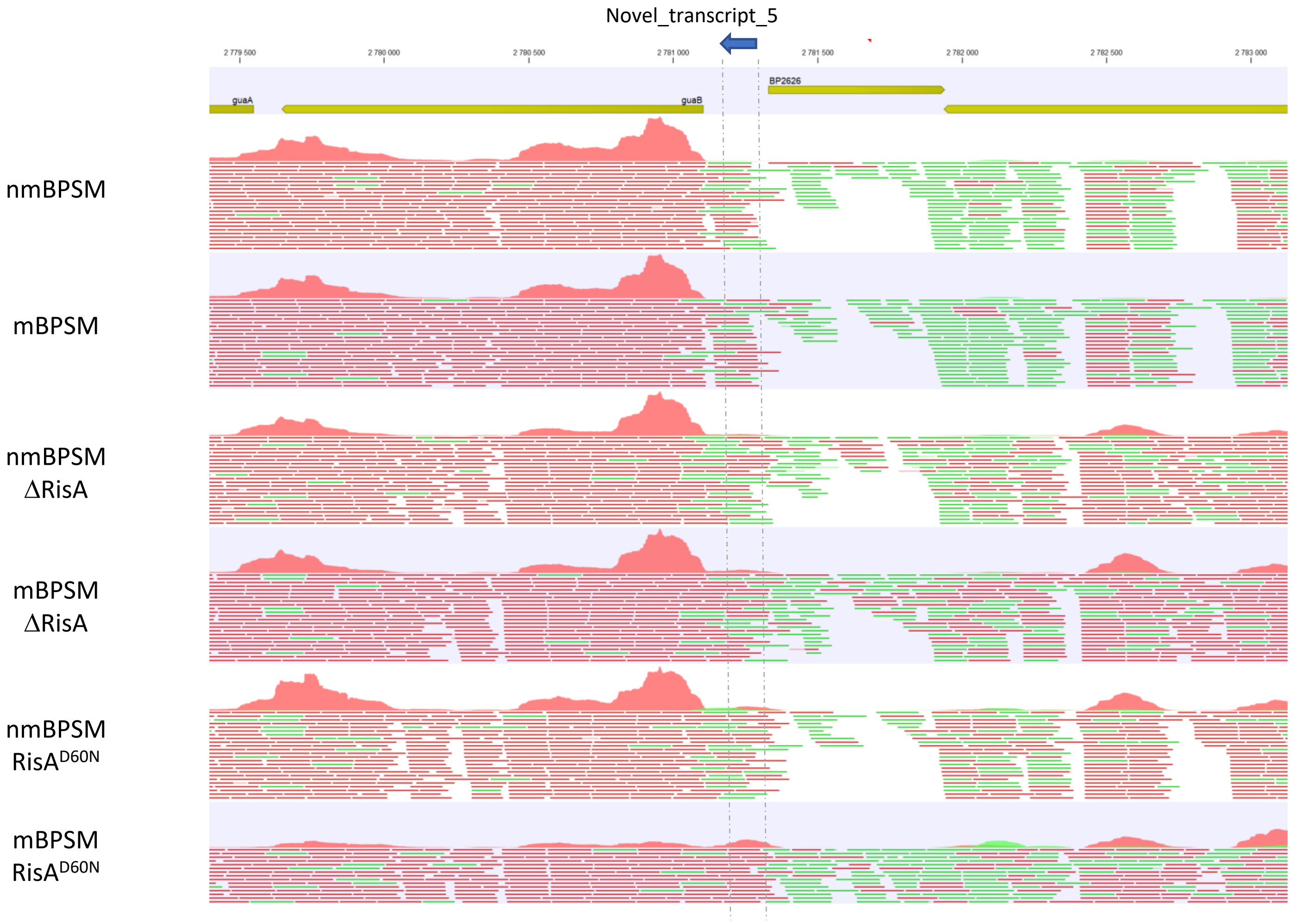

Figure S9. Localization of Novel_transcript_5 a putative sRNA regulated by RisA. Schematic representation of the genetic environment of the sRNA with the adjacent ORFs in the *B. pertussis* Tohama I BX470248 genome. The respective transcripts and the orientation of their expression are indicated by the blue arrows, while the surrounding ORFs and their orientation are indicated by the yellow arrows. bp numbers correspond to the genome coordinates as annotated in the *B. pertussis* Tohama I BX470248 genome. Single reads mapping in the forward direction are in green. Single reads mapping in the reverse direction are red

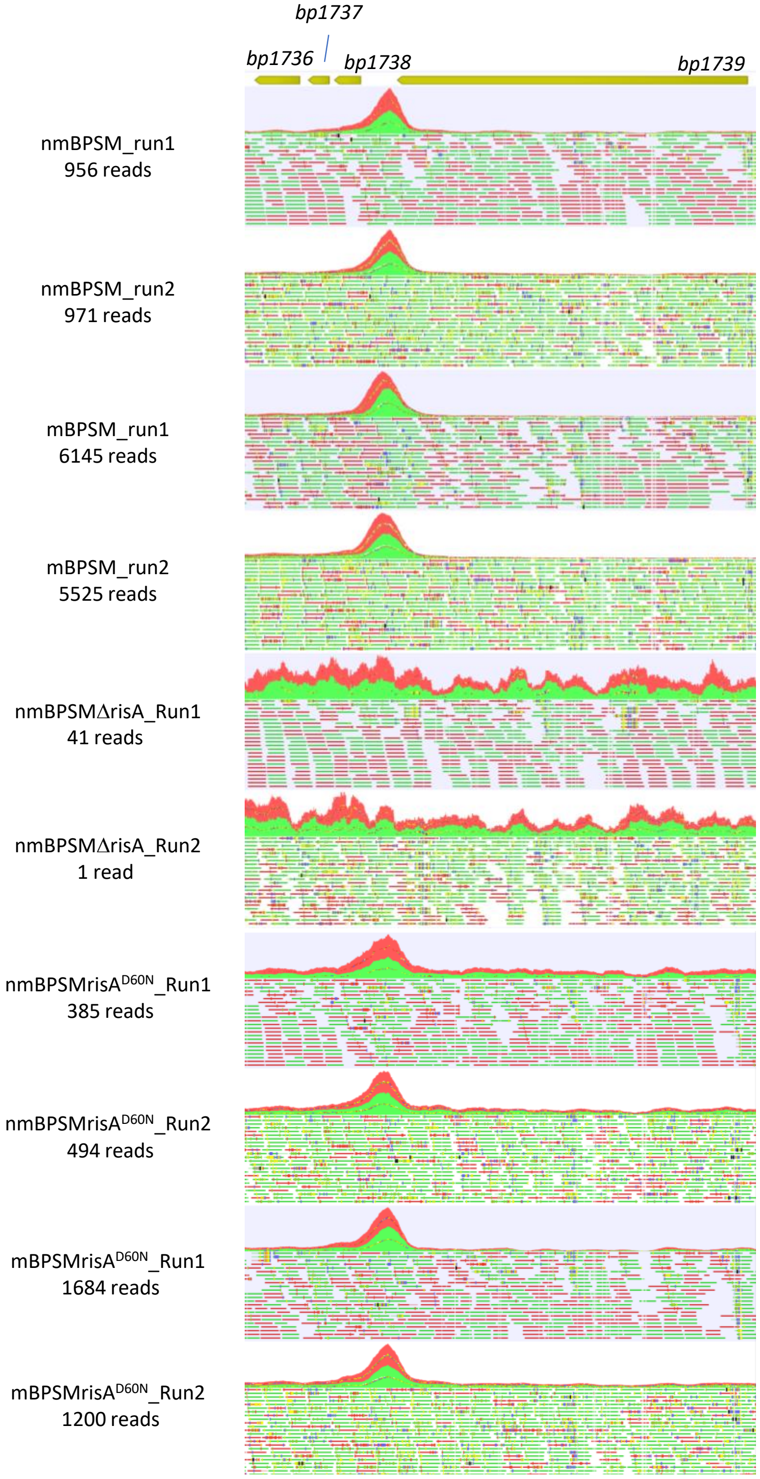

Figure S10. Screen shot representation of the read mapping of the ChIPseq results in the *bp1738* promoter. ORFs and their orientation are indicated by the yellow arrows. Single reads mapping in the forward direction are in green. Single reads mapping in the reverse direction are red. Read mapping was done using CLC genomic workbench 23. Read numbers correspond to the amount of reads detected by the depth module of SAMtools at the center of the detected peak in each condition.

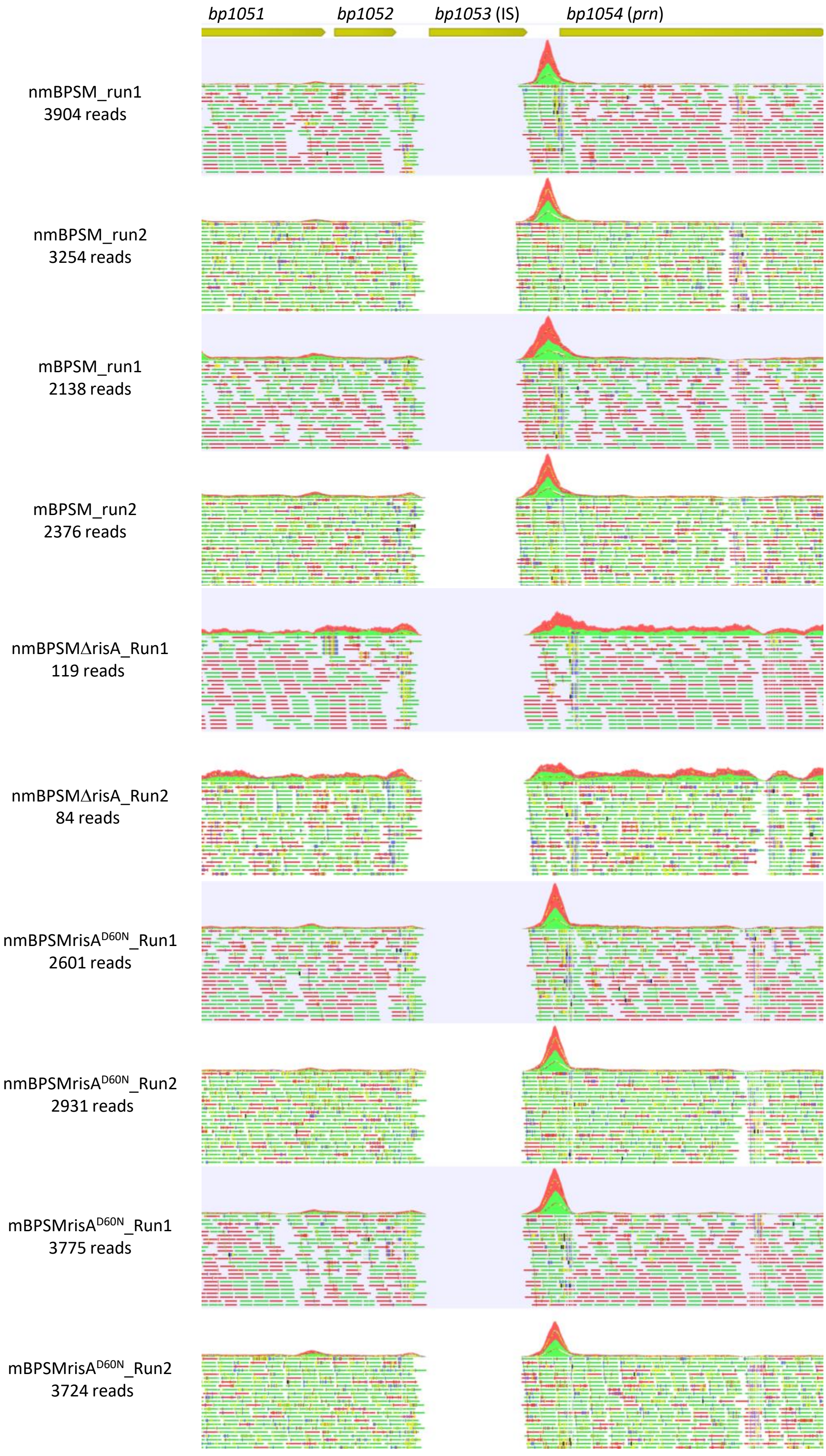

Figure S11. Screen shot representation of the read mapping of the ChIPseq results in the *prn* promoter. ORFs and their orientation are indicated by the yellow arrows. Single reads mapping in the forward direction are in green. Single reads mapping in the reverse direction are red. Read mapping was done using CLC genomic workbench 23. Read numbers correspond to the amount of reads detected by the depth module of SAMtools at the center of the detected peak in each condition.

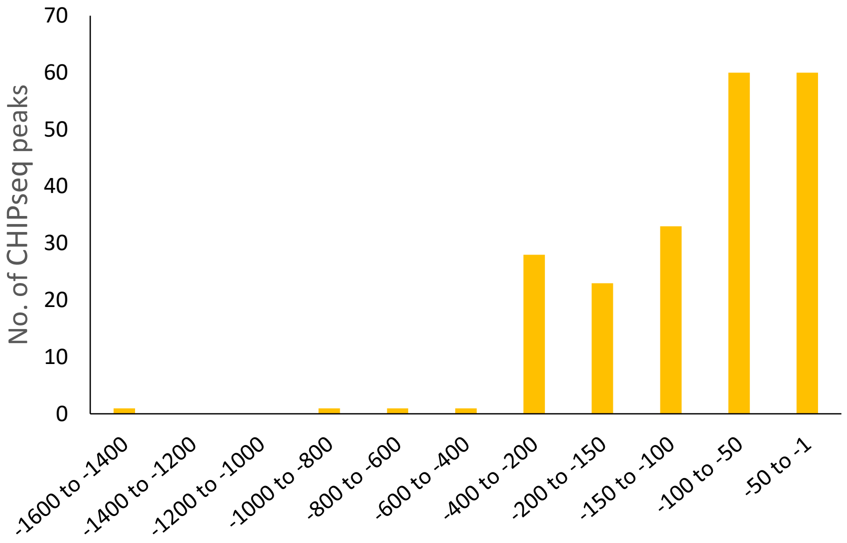

Figure S12. Localization of the RisA-binding sites within in promoter regions. Number of ChIPseq peaks are depicted according to the distance in nucleotides between the center of the peak and the predicted ATG translational start site of the corresponding ORF

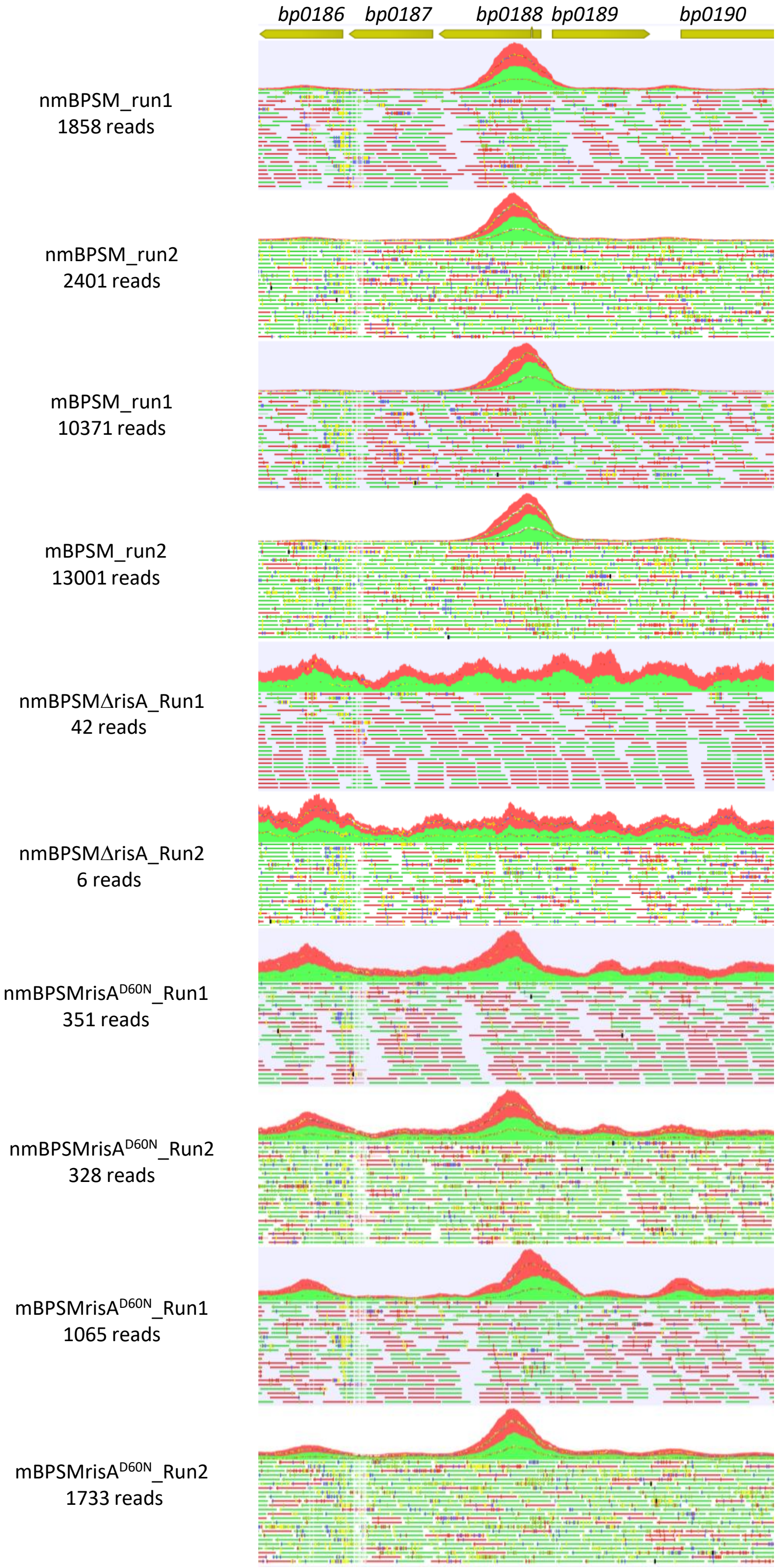

Figure S13. Screen shot representation of the read mapping of the ChIPseq results within *bp0188*. ORFs and their orientation are indicated by the yellow arrows. Single reads mapping in the forward direction are in green. Single reads mapping in the reverse direction are red. Read mapping was done using CLC genomic workbench 23. Read numbers correspond to the amount of reads detected by the depth module of SAMtools at the center of the detected peak in each condition.

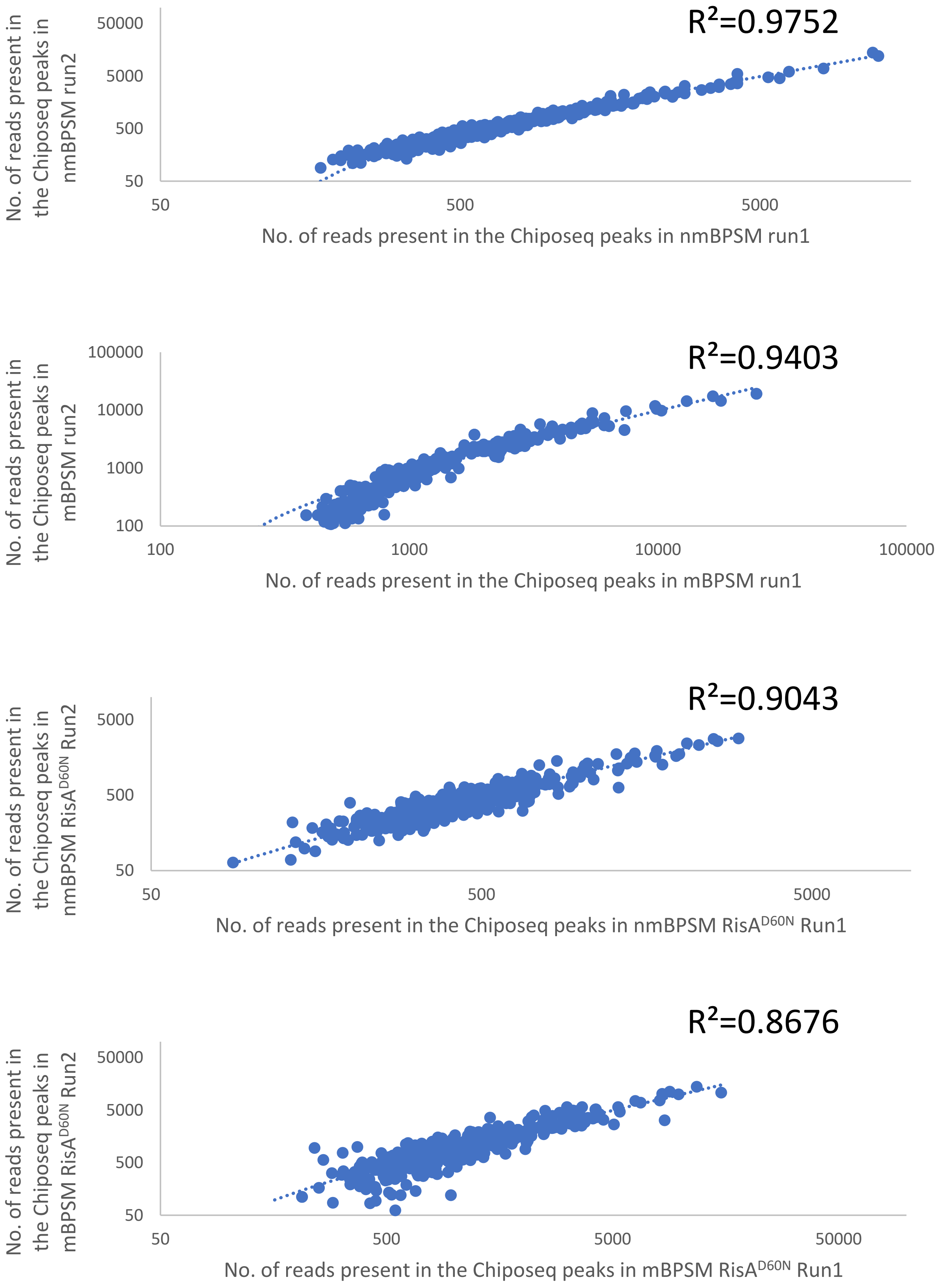

Figure S14. Scatter-plots representation of the ChIPeq data between two runs corresponding to the same conditions. The regression curve and the R^2 are specified on each graph.

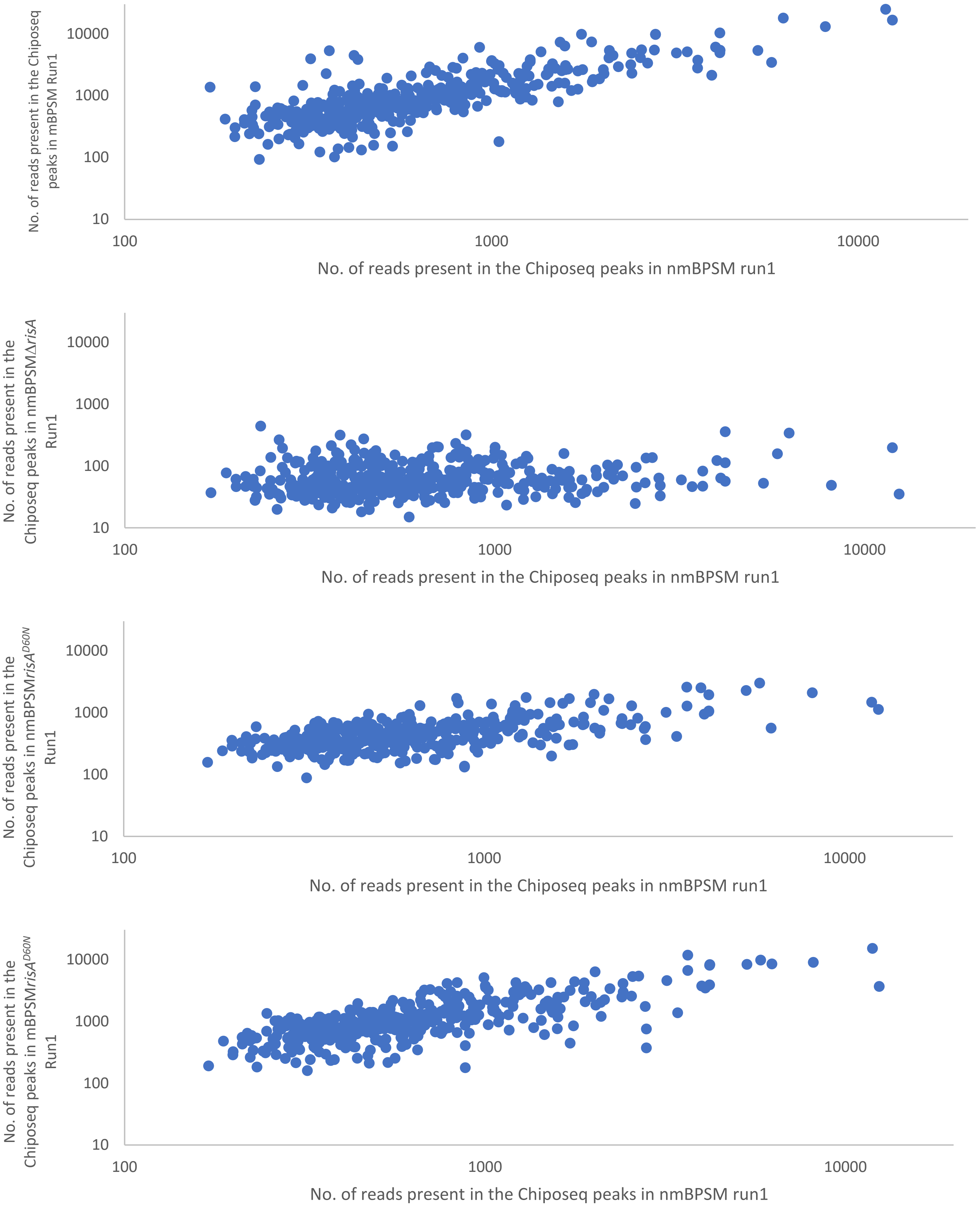

Figure S15. Scatter-plots representation of the ChIPseq peaks data between conditions.
The number of reads at the center of each ChIPseq detected peak is presented for each condition. (A) nmBPSM run1 vs mBPSM run1, (B) nmBPSM run1 vs nmBPSMΔ*risA* run1, (C) nmBPSM run1 vs nmBPSM*risA*^{D60N} and (D) nmBPSM run1 vs mBPSM*risA*^{D60N} .
